# Modeling Factors Associated with Smoking Dependence Severity Using Binary Classification-Based Machine Learning: Prediction with Hematological and Demographic Patient Data

**DOI:** 10.3390/diagnostics16203425

**Published:** 2026-10-21

**Authors:** Yavuz Korkmaz, İbrahim Miraç Eligüzel, Adem Taha Özdemir

**Affiliations:** 1 Department of Family Medicine, Malatya Turgut Özal University, Malatya 44210, Türkiye; adem.ozdemir@ozal.edu.tr; 2 Department of Industrial Engineering, Gaziantep Islam Science and Technology University, Gaziantep 27010, Türkiye; ibrahim.eliguzel@gibtu.edu.tr

**Keywords:** smoking dependence, psychological dependence, nicotine dependence, machine learning, hematological parameters

## Abstract

**Background/Objectives**: The Fagerström Test for Nicotine Dependence (FTND) is a subjective, self-report-based instrument used to grade the level of nicotine dependence. Complete blood count (CBC) parameters reflect markers of smoking-related systemic inflammation. In the present study, we aimed to develop a leakage-free and interpretable machine learning approach for classifying FTND level using demographic data, CBC parameters, and duration of smoking exposure. **Methods**: In this retrospectively designed study, the records of 660 patients with complete data who had presented to the Smoking Cessation Outpatient Clinic of Malatya Training and Research Hospital were included. Because one of the six FTND items directly scores the number of cigarettes smoked per day, the number of packs and the features related to it constitute data leakage and were therefore excluded from the data pool. Smoking duration (years), which is not an FTND item, was retained as the sole predictor of smoking exposure. Feature engineering generated 49 candidate features. Six base learners (logistic regression, random forest, gradient boosting, SVM, LightGBM, XGBoost) and a stacking ensemble were evaluated with an 80–20% training/test split and 10-fold stratified cross-validation. To prevent optimism bias, feature selection and scaling were performed only within the training folds, and class imbalance was addressed through class weighting. The models were interpreted with SHAP and LIME, and the contribution of each feature family was quantified by ablation analysis. **Results**: In leakage-free 10-fold cross-validation (the primary performance estimate), discrimination was modest, with the highest AUC (0.652) obtained by both SVM (RBF) and logistic regression; on the single hold-out test set, the stacking ensemble reached an AUC of 0.749 (95% CI 0.667–0.831). The full model did not significantly outperform smoking duration alone (DeLong *p* = 0.22), and extensive feature engineering did not provide a measurable gain (ΔAUC = −0.015). In the SHAP analysis, smoking duration and the age-based interaction terms made the largest predictive contributions. **Conclusions**: Routine CBC parameters do not by themselves constitute an independent diagnostic tool for determining dependence severity; they carry only weak, complementary information and should not be regarded as a screening biomarker. This exploratory study classifies FTND-based nicotine dependence severity at the individual patient level by combining CBC parameters with machine learning and explainable artificial intelligence; the model is not ready for clinical use.

## 1. Introduction

According to the World Health Organization, smoking causes more than seven million deaths every year and is counted among the leading causes of preventable disease and death worldwide [1]. The level of nicotine dependence is an important factor that can directly affect both the success of smoking cessation treatment and the treatment to be applied. For this reason, accurate and reliable assessment of dependence is important for an effective treatment plan.

The Fagerström Test for Nicotine Dependence (FTND), one of the most widely used instruments for measuring nicotine dependence, makes it possible to assess the level of physical dependence rapidly and in a form applicable in the clinical setting. The scale was revised in 1991 in order to address the psychometrically criticized aspects of the Fagerström Tolerance Questionnaire (FTQ). In this revision, two items that did not show sufficient association with the biochemical indicators of heavy smoking (the nicotine yield of the cigarette and inhalation of the smoke) were removed from the scale; in contrast, the scoring of the items on time to the first cigarette in the morning and the number of cigarettes consumed per day was converted into a four-point rating, thereby increasing discrimination. These changes improved the agreement of the scale’s dependence severity with biochemical criteria such as cotinine level and made the FTND more widely used both in clinical assessment and in epidemiological research [2]. The Turkish adaptation of the scale and the testing of its reliability were carried out by Uysal et al. (2004); in a sample of 169 smokers, the internal consistency of the Turkish form was found to be moderate (Cronbach’s alpha = 0.56) [3].

Bergen et al. predicted nicotine biomarkers in a multiethnic sample and, by showing that these markers were associated with nicotine dependence, indicated that measurable biochemistry can be used in the assessment of dependence [4]. Cui et al., in turn, reported that inter-individual variability in brain connectivity predicted nicotine dependence severity through machine learning and differentiated smokers [5]. Similarly, Çay et al. achieved the detection of cigarette addiction by applying deep learning to electroencephalography data [6]. In the field of neuroimaging, Smith et al. analyzed the functional connectivity of resting-state functional magnetic resonance imaging networks in nicotine-dependent individuals [7].

In studies aimed at the alternative assessment of nicotine dependence, machine learning methods have been found to yield successful results using magnetic resonance imaging (MRI), electroencephalography (EEG) and various physiological signals [5,6,7,8]. However, because most of these methods are costly, require special equipment and are not easily applicable in routine health services, they have been found unsuitable for daily clinical use [9]. For this reason, there is a need for objective assessment methods that can be developed from easily accessible, low-cost clinical data obtained routinely.

Such an objective indicator would not replace the FTND, which is itself inexpensive and easy to administer, but it could add value in situations in which the FTND is unavailable or may be unreliable: in retrospective analyses of routinely collected clinical data in which the FTND has not been recorded, in clinical settings outside dedicated smoking-cessation services where dependence is not formally assessed, and as an objective complement to a self-reported score that is susceptible to recall and social-desirability bias. Whether routinely available laboratory data carry enough information for such uses has not, however, been quantified with a design that excludes the variables overlapping with the FTND.

The complete blood count is a candidate data source worth evaluating for this purpose because of its low cost, widespread use and place among routine laboratory investigations. Chronic inflammation and hematological changes that may develop in relation to smoking can be reflected in CBC parameters. Existing studies show that smoking causes changes in the neutrophil-to-lymphocyte ratio (NLR), the platelet-to-lymphocyte ratio (PLR), the mean platelet volume (MPV) and other platelet parameters [10,11]. In addition, marked improvements in these inflammatory indicators have been observed following smoking cessation [12]. Differing from these studies, Güden et al., in a sample of 247 smokers aged 18–40 years without comorbidity, found the NLR and PLR values of individuals with a high dependence level to be significantly higher than those of the others, and reported that both ratios showed a positive correlation with the FTND score, the number of cigarettes per day and the exhaled CO level, and a negative correlation with the lymphocyte-to-monocyte ratio (LMR) [13].

Cumulative smoking exposure is one of the most widely used measures for assessing long-term exposure to tobacco smoke and is usually expressed as pack-years (packs per day × years). However, one of the six FTND items directly scores the number of cigarettes smoked per day and contributes up to 3 of the total 10 points. For this reason, the number of cigarettes/packs per day and the pack-year variable derived from it are not independent predictors but components of the outcome; their use in predicting the level of dependence leads to target leakage. In contrast, smoking duration (years), which is not one of the FTND items, can be used as a legitimate measure of cumulative exposure.

Machine learning methods have made it possible to evaluate linear and non-linear relationships among a large number of clinical and laboratory variables. For this reason, the use of machine learning methods in biomedical research is steadily increasing. In the field of smoking, these methods have been applied for various purposes such as determining smoking status, assessing nicotine dependence, and predicting smoking cessation success and relapse risk, and these studies have yielded successful results [14,15,16].

Previous machine-learning studies of nicotine dependence have relied on questionnaire-based, behavioral and sociodemographic predictors rather than laboratory data: Bulut et al. modeled the FTND score as a continuous outcome in 1120 university students (LASSO, R^2^ = 0.62) [14], Etter et al. predicted smoking cessation and relapse in 5293 daily smokers [15], and Kharabsheh et al. classified waterpipe dependence into three levels using 19 sociodemographic and behavioral variables [16].

Studies in which the level of nicotine dependence assessed with the FTND is evaluated using CBC parameters and duration of smoking exposure together are, however, very limited. Moreover, most of the research in this field has been conducted with classical statistical methods, and insufficient use has been made of machine learning approaches capable of evaluating the complex relationships among numerous hematological variables. In the present study, we aimed to develop a leakage-free and interpretable machine learning approach for classifying FTND level using demographic data, hemogram parameters and duration of smoking exposure (years).

## 2. Materials and Methods

### 2.1. Overview and Study Design

In this study, we aimed to develop a machine learning approach for classifying FTND level using routinely obtainable CBC parameters together with basic demographic and smoking exposure information. Unlike physiological signal, neuroimaging or electrophysiological approaches, the CBC is a low-cost, minimally invasive and almost universally accessible laboratory test. These characteristics make it worthwhile to examine whether the CBC carries information on dependence severity that could complement existing assessment instruments. To this end, a supervised machine learning pipeline was developed in which feature engineering, feature selection and preprocessing were all performed within the cross-validation training folds, so that information leakage was prevented and generalizable performance estimates were obtained. This study is presented as a proof-of-concept rather than as a clinically validated diagnostic system.

**Figure 1:**
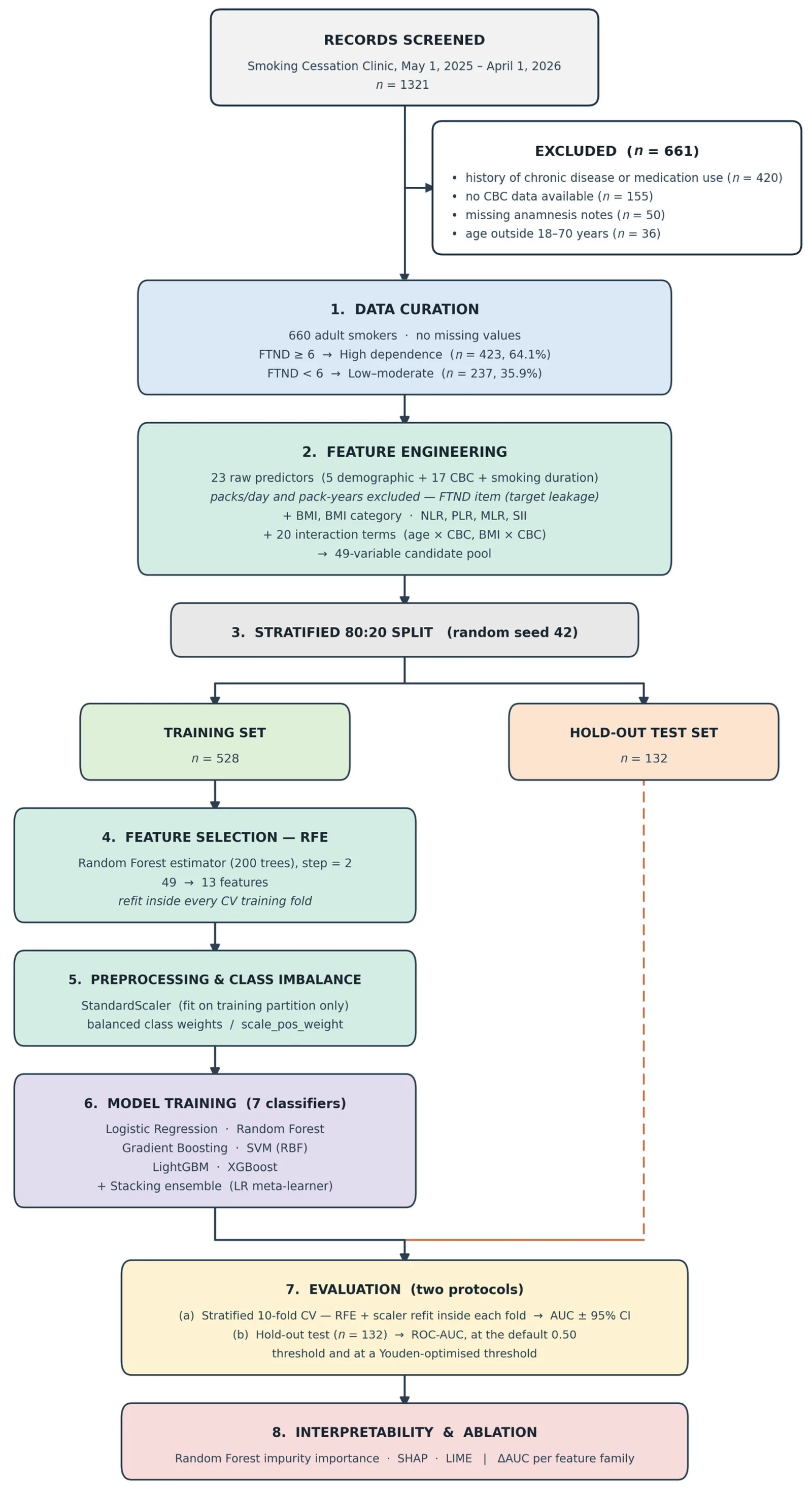
Overview of the study workflow, including the participant flow from the 1321 screened records to the 660 analyzed patients. The hold-out test set (*n* = 132) was separated before feature selection and scaling and was used only for final evaluation, ensuring leakage-free performance estimates. Solid arrows indicate the sequence of the pipeline; the dashed line indicates that the hold-out test set was used only in the final evaluation; box colors distinguish the stages of the workflow. RFE, recursive feature elimination; CBC, complete blood count; CV, cross-validation.

The overall workflow (Figure 1) consists of the following stages: data curation, domain knowledge-based feature engineering, leakage-free feature selection with Recursive Feature Elimination (RFE), model training in which class imbalance is explicitly addressed, evaluation with both a hold-out test set and stratified cross-validation, model interpretation using SHAP and LIME, and an ablation analysis quantifying the contribution of each feature family.

### 2.2. Study Population and Dataset

Between 1 May 2025 and 1 April 2026, the medical records of 1321 patients who presented to our Smoking Cessation Clinic were retrospectively reviewed. Of these, 660 patients aged 18–70 years who had complete data and no history of chronic disease or medication use were included in the study. The remaining 661 patients were excluded for the following reasons: a history of chronic disease or medication use (*n* = 420), no CBC data available (*n* = 155), missing anamnesis notes (*n* = 50) and age outside the 18–70-year range (*n* = 36) (Figure 1). All participants were smokers with no known chronic disease and no regular medication use; this minimizes the confounding of hematological parameters by comorbidities or pharmacological effects. The mean age was 35.4 ± 10.8 years (range 18–70), and our study comprised 449 men (68.0%) and 211 women (32.0%). The mean body mass index (BMI) was calculated as 25.0 ± 3.7 kg/m^2^. Reported smoking exposure was a mean of 1.19 ± 0.49 packs per day over a mean period of 14.8 ± 9.8 years (range 1–45). For each participant, seven demographic and clinical variables, smoking history (number of cigarettes per day and duration in years), the total FTND score and a 17-parameter CBC panel (measured with a DH-800 automated hematology analyzer; Shenzhen Dymind Biotechnology Co., Ltd., Shenzhen, China) were recorded. There were no missing values in the dataset.

### 2.3. Ethics Approval and Consent to Participate

This study was approved by the Scientific Research Ethics Committee for Health Sciences of Malatya Turgut Özal University (Malatya, Türkiye; application no. 2026/219 dated 18 May 2026, found ethically appropriate unanimously at session no. 24 dated 21 May 2026; decision letter no. E-30785963-020-405632 dated 13 July 2026). All methods were performed in accordance with the relevant guidelines and regulations, including the Declaration of Helsinki. Owing to the retrospective design of the study and the use of anonymized routinely collected clinical data, the requirement for individual informed consent was waived by the Scientific Research Ethics Committee for Health Sciences of Malatya Turgut Özal University. All patient records were fully anonymized before analysis, and no personally identifiable information was accessible to the investigators.

### 2.4. Outcome Definition and Class Labeling

The FTND is a six-item self-report scale derived from the Fagerström Tolerance Questionnaire and revised by Heatherton et al. [2]; the item scores are summed to give a total score ranging from 0 to 10, with higher values indicating higher physical nicotine dependence. In the present cohort, FTND scores were spread across the entire 0–10 scale. In order to create a clinically meaningful and statistically robust target variable, participants were dichotomized at a threshold of 6 points: those with FTND ≥ 6 were labeled high dependence (*n* = 423, 64.1%), and those with FTND < 6 were labeled low-to-moderate dependence (*n* = 237, 35.9%). The cut-off of 6 is a widely used threshold for defining high nicotine dependence [2,3]. This produced a moderately imbalanced binary classification problem, which was explicitly addressed during modeling (see ‘Data preprocessing and handling of class imbalance’). A binary rather than an ordinal or multi-class formulation was chosen because the cut-off corresponds directly to a clinical decision: in the routine practice of our smoking cessation clinic, management is decided on a binary high-versus-low basis, essentially whether pharmacological support should be intensified, rather than on the full ordinal score, so the binary formulation matches the way the scale is actually used in this setting. In addition, modeling the full 0–10 scale as an ordinal or multi-class outcome would have divided the 660 participants into small and markedly imbalanced subgroups (several score levels contained fewer than 40 patients), reducing statistical power and yielding clinically ambiguous outputs. Two sensitivity analyses of the outcome definition were performed with the same leakage-free protocol: the analysis was repeated at the alternative cut-offs FTND ≥ 5 and ≥ 7, and the FTND score (0–10) was modeled as a continuous outcome (linear regression and random-forest regression, with RFE guided by a random-forest regressor and refitted within each fold). For the continuous outcome, predictive accuracy was summarized by the mean absolute error (MAE), the root mean squared error (RMSE) and the coefficient of determination (R^2^) of the pooled out-of-fold predictions, compared with a reference model predicting the training-fold mean. Importantly, one of the six FTND items scores the number of cigarettes smoked per day and contributes up to 3 of the total 10 points. Consequently, the recorded number of cigarette packs per day is not an independent predictor but a component of the outcome; every variable derived from it, including the pack × year product, carries the same target leakage. For this reason, packs per day and pack × year were excluded a priori from the candidate feature pool, in line with established guidance on target leakage [17]. Smoking duration (years), which is not an FTND item, was retained in the pool as the sole predictor of smoking exposure.

### 2.5. Feature Engineering

Consistent with the cohort’s inclusion criteria, the recorded indicators of comorbidity and medication use were uniformly zero in all participants and therefore carried no variance; both were excluded a priori. Beyond the remaining 23 raw predictors (5 demographic variables, 17 CBC parameters and smoking duration), a set of domain knowledge-based features was derived, expanding the candidate pool to 49 variables (Table 1). All derived features were computed from the raw variables as described below.

**Smoking exposure.** Cumulative tobacco exposure was represented by smoking duration in years. Among the recorded smoking variables, this is the only one that is not a component of the FTND (see ‘Outcome definition and class labeling’), and it was included directly in the model without any transformation.

**Anthropometry.** Body mass index was calculated from height and weight using the formula ‘Weight (kg)/Height^2^ (m^2^)’ and was additionally discretized into the four categories of the World Health Organization (<18.5; 18.5–24.9; 25.0–29.9; and ≥30 kg/m^2^), coded as 0–3 respectively.

**Systemic inflammation indices.** Because chronic smoking is associated with subclinical systemic inflammation, four hematological ratios established in the literature were derived from the CBC panel: the neutrophil-to-lymphocyte ratio (NLR), the platelet-to-lymphocyte ratio (PLR), the monocyte-to-lymphocyte ratio (MLR) and the systemic immune-inflammation index [18]:

NLR = NEU/LYM(1)

PLR = PLT/LYM(2)

MLR = MONO/LYM(3)

SII = PLT × NEU/LYM(4)

**Interaction terms.** In order to capture the age-dependent modulation of the hematological profile, first-order interaction terms were created between age and each of the 17 CBC parameters (age × CBC). In addition, three hematological terms scaled by BMI were included (BMI × HGB, BMI × WBC and BMI × PLT). The full composition of the resulting 49-variable pool is summarized in Table 1.

### 2.6. Feature Selection

In order to keep the model parsimonious and, more importantly, to let the data rather than the researchers decide which variables were relevant, Recursive Feature Elimination was applied to reduce the 49-variable pool to 13 features [19]. The number of features was fixed a priori rather than optimized on the data, so that no additional data-dependent choice entered the cross-validated estimates; RFE determined which features were retained. With 13 features, the minority (Low dependence) class provides 14.6 events per variable in the training set and 13.2 in each cross-validation training partition, above the commonly recommended minimum of 10 events per variable [20]; this criterion would be met by any number of features up to 17, and at the upper end of this range (11 to 17 features) performance varied little (Section 3.3). RFE was guided by a Random Forest estimator (200 trees, balanced class weights) with a step size that eliminated two features at each iteration. Critically, RFE was retrained inside each cross-validation training fold, so that no information from the hold-out data influenced feature selection.

In order to characterize the stability of the selection, the number of folds out of ten in which each variable was retained was recorded (selection frequency analysis). Five variables, smoking duration, BMI, SII, age × PLT and age × MCV, were retained in all ten folds, and one variable (age × EOS) in nine, indicating a consistent and data-driven association. The 13 features selected in the training set were as follows: smoking duration, PLT, BMI, and the interaction terms age × WBC, age × PLT, age × NEU, age × LYM, age × BASO, age × EOS, age × MCV, age × PCT, BMI × HGB and BMI × PLT.

### 2.7. Data Preprocessing and Handling of Class Imbalance

Within each training fold, the selected features were standardized to zero mean and unit variance (z-score normalization); the scaler was fitted only on the training portion and then applied to the hold-out data, thereby preventing leakage of test set statistics. Instead of synthetic minority oversampling, the moderate class imbalance was addressed at the algorithm level through cost-sensitive learning, in which each class was assigned a weight inversely proportional to its frequency:

w_j_ = N/(C × N_j_) (5)

Here N is the total number of training samples, C the number of classes, and N_j_ the number of samples in class j. By increasing the weight of the minority class (low-to-moderate dependence) within the loss function, these weights prevent the classifier from being biased toward the majority class.

### 2.8. Machine Learning Models

Seven classifiers were trained and compared: (i) L2-regularized Logistic Regression, (ii) Support Vector Machine (SVM) with a radial basis function kernel, (iii) Random Forest, (iv) Gradient Boosting, (v) XGBoost, (vi) LightGBM and (vii) a Stacking ensemble combining the base learners through a logistic regression meta-learner. Tree-based ensembles and two gradient boosting implementations were included in order to capture non-linear feature interactions, whereas Logistic Regression provided an interpretable linear baseline model [21,22]. Balanced class weights were used wherever supported by the algorithm. No hyperparameter search was performed; each model was trained with fixed, predetermined hyperparameters and a common random seed of 42. This choice was deliberate. Leakage-free tuning would have required a further inner optimization loop within each outer cross-validation fold; with only 52–53 participants per validation fold (19 in the Low class), the selected values would have been unstable and any gain uncertain, whereas conventional fixed settings keep the pipeline fully reproducible. Logistic Regression used an L2 penalty with the default regularization strength (C = 1.0) and the lbfgs solver (maximum 2000 iterations). SVM used a radial basis function kernel with C = 1.0 and the default kernel coefficient (γ = ‘scale’, that is, 1/[number of features × Var(X)]). Random Forest consisted of 300 trees grown to full depth with the Gini criterion, with all other settings left at the scikit-learn defaults. Gradient Boosting used 300 estimators, a learning rate of 0.05 and a maximum tree depth of 4. XGBoost similarly used 300 estimators, a learning rate of 0.05 and a maximum depth of 4, with the log-loss evaluation metric. LightGBM used 300 estimators, a learning rate of 0.05 and a maximum depth of 4. The balanced class weighting described above was applied to Logistic Regression, SVM, Random Forest and LightGBM; Gradient Boosting does not support class weighting, whereas XGBoost handles class imbalance through the scale_pos_weight parameter, which was set to the ratio of negative to positive samples. The stacking ensemble used these six models as base learners: their out-of-fold class probability predictions, generated by an inner stratified 10-fold cross-validation, were passed to an L2-regularized Logistic Regression meta-learner (C = 1.0, maximum 2000 iterations, balanced class weights).

### 2.9. Model Training, Validation and Evaluation

Two complementary evaluation protocols were applied. First, the cohort was split into a training set and a hold-out test set: an 80:20 ratio (test_size = 0.20), stratification by dependence class and a fixed random seed (random_state = 42) yielded 528 training and 132 hold-out test participants (training set: 338 High and 190 Low; hold-out test set: 85 High and 47 Low); the final receiver operating characteristic (ROC) curves and the area under the curve (AUC) on unseen data were obtained on this test set, whereas the operating decision threshold was selected on the training data by 10-fold cross-validation (from out-of-fold predictions) and then applied unchanged to the hold-out test set. Second, stratified 10-fold cross-validation (with the data shuffled using a fixed random seed) was applied in order to estimate the stability of performance and to compute 95% confidence intervals. The cross-validation was performed within the training set (*n* = 528): each of the ten validation folds contained 52–53 participants (33–34 High and 19 Low), and each corresponding training partition contained 475–476 participants (304–305 High and 171 Low). To assess the stability of these estimates, the leakage-free 10-fold cross-validation was repeated with ten different random partitions of the training set (10 × 10-fold) for logistic regression and SVM. In both protocols, feature selection and standardization were restricted to the training data only, thereby ensuring leakage-free estimates.

Discrimination was assessed primarily with the AUC, a threshold-independent metric. Threshold-dependent metrics, accuracy, sensitivity (recall), specificity, precision and F1 score, were computed both at the default probability threshold of 0.50 and at a threshold optimized to balance sensitivity and specificity.

Calibration, that is, the agreement between predicted probabilities and observed proportions, was assessed on the hold-out test set. The Brier score [23], the mean squared difference between the predicted probability and the observed outcome (lower values are better), was compared with that of a non-informative model assigning the training-set prevalence of high dependence to every patient. The calibration intercept (calibration-in-the-large; ideal value 0) and the calibration slope (ideal value 1) were estimated by logistic regression of the observed outcome on the logit of the predicted probability, the intercept with the logit entered as an offset. In calibration plots, the observed proportions were plotted against the mean predicted probabilities across quintiles of predicted probability, together with the logistic calibration curve. As a robustness check, the same measures were computed from the pooled out-of-fold predictions of the leakage-free 10-fold cross-validation (*n* = 528).

To obtain leakage-free cross-validated estimates, all data-dependent steps were nested inside the cross-validation. In each of the ten outer folds, (i) recursive feature elimination (RFE with a 200-tree random-forest estimator, selecting 13 features) was fitted on the outer-training partition only; (ii) the StandardScaler was fitted on the same outer-training partition and applied to the held-out fold; and (iii) every classifier, the six base learners and the stacking ensemble, was trained exclusively on the outer-training partition and evaluated on the untouched outer-validation fold. Class weighting (class_weight = ‘balanced’; scale_pos_weight for XGBoost) was specified in each estimator and therefore applied at every fit. For the stacking ensemble, the level-0 out-of-fold predictions used to train the logistic-regression meta-learner were generated by an inner stratified 10-fold cross-validation performed entirely within the outer-training partition; the outer-validation fold contributed to none of these steps. Feature selection and scaling were fixed per outer fold and were not re-estimated within the stacking’s inner folds; because the outer-validation fold was excluded from feature selection, scaling and all model fitting, the reported cross-validated AUCs are not optimistically biased by the ensemble. A sensitivity analysis with RFE and scaling re-fitted within the inner folds gave virtually identical results (Section 3.1).

### 2.10. Model Interpretability

In order to move beyond black-box prediction and to describe which features the model relies on for its classifications, three complementary explainability techniques were applied. SHAP and LIME were computed for the logistic regression model, which shared the highest cross-validated AUC and, being linear, allows the contribution of each feature to be attributed exactly. The impurity-based importance measures of the Random Forest provided a global ranking of predictor contributions. SHapley Additive exPlanations (SHAP) [24] quantified both the mean magnitude and the direction of the contribution of each feature to the model output; these results were summarized with mean absolute SHAP values and beeswarm plots. Local Interpretable Model-agnostic Explanations (LIME) [25] were generated for individual participants in order to explain individual predictions, that is, to show which features contributed most to the prediction for a single patient. These methods describe how the fitted model uses its inputs (predictive contributions); they do not establish biological or causal effects. Because several engineered features are strongly correlated with one another and with their component variables, the contribution attributed to an individual feature, including its sign, should not be interpreted physiologically.

Multicollinearity among the 13 selected features was assessed in the training set (*n* = 528) with pairwise Pearson correlation coefficients and variance inflation factors (VIF; values above 10 were taken to indicate strong collinearity). The direction of the univariate association of each feature with the FTND score was assessed with Spearman’s rank correlation, and the combined contribution of correlated features was computed as the mean absolute value of the sum of their SHAP values.

### 2.11. Ablation Analysis

In order to quantify the net predictive contribution of each feature family, and to verify that classification performance was not an artifact of the derived features, an ablation study was conducted. Starting from the full 49-feature pool, feature groups were removed one by one (interaction terms; derived indices; all derived features together; and smoking duration), and the leakage-free 10-fold cross-validated AUC was recomputed for each configuration. Three reference configurations were additionally evaluated: smoking duration only, raw CBC only, and demographic variables together with raw CBC without any derived features. For each configuration, the change in AUC relative to the full pool (ΔAUC) was used as a measure of the contribution of the corresponding group.

### 2.12. Statistical Analysis

The association of the variables with the level of dependence was also examined with classical statistical methods, independently of the machine learning models. The distribution of the variables was assessed with the Shapiro–Wilk test, and all continuous variables examined, including the Fagerström score, were found not to be normally distributed (*p* < 0.001 for all). Because the Fagerström score is an ordinal variable ranging from 0 to 10, the associations were tested with non-parametric methods. Throughout the manuscript, “association” refers to these univariate, group-level relationships with the FTND score, whereas “prediction” refers to the out-of-sample classification of individual patients by the machine-learning models (Section 2.8 and Section 2.9); a statistically significant association does not by itself imply useful individual-level prediction.

The univariate association between the continuous Fagerström score and each variable was assessed with the Spearman rank correlation coefficient (ρ). In addition, differences between the High (≥6) and Low (<6) dependence groups were compared with the Mann–Whitney U test, and effect size was measured with the rank-biserial correlation. Because a total of 27 variables were tested, the Benjamini–Hochberg false discovery rate (FDR) correction was applied in order to control the risk of false positives arising from multiple comparisons, and significance was interpreted according to an FDR-corrected threshold of *p* < 0.05.

Pairwise differences in AUC between the three main feature configurations (full pool, smoking duration alone and raw CBC alone) were assessed with DeLong’s test [26] for two correlated ROC curves, applied to the pooled out-of-fold predictions of the leakage-free 10-fold cross-validation (training set, *n* = 528; each participant contributes exactly one prediction per configuration; folds, feature selection and model as in the ablation analysis); two-sided 95% confidence intervals for the AUC differences were obtained by stratified bootstrap resampling (3000 replicates). For the hold-out test AUC (*n* = 132; 85 High and 47 Low), 95% confidence intervals were computed with the Hanley–McNeil method [27]; a percentile bootstrap with 2000 stratified resamples gave intervals of essentially the same width. For the cross-validated AUC, 95% confidence intervals were obtained across the ten folds as the mean ± 1.96·SD/√10. For the threshold-dependent metrics on the hold-out test set, 95% confidence intervals were computed with the Wilson score method [28] for sensitivity, specificity and accuracy, and with a percentile bootstrap (10,000 resamples stratified by class, with the decision threshold held fixed) for the F1 score. To examine whether smoking duration reflects age or cumulative exposure, partial Spearman correlations of the FTND score with smoking duration and with age, each adjusted for the other, were computed, and logistic regression models containing age, smoking duration or both were compared in the leakage-free cross-validation of the training set with DeLong’s test.

### 2.13. Implementation

All analyses were performed in Python using the scikit-learn library together with the xgboost, lightgbm, shap and lime packages. A fixed random seed was used throughout in order to ensure reproducibility. The versions used were as follows: Python 3.12.3, scikit-learn 1.9.0, XGBoost 3.0.5, LightGBM 4.6.0, SHAP 0.48.0, LIME 0.2.0.1, NumPy 2.2.6, pandas 2.3.3, Matplotlib 3.8.4, seaborn 0.13.2 and openpyxl 3.1.4 [29]. All analyses were carried out on a workstation with a 12th-generation Intel Core i7-1255U processor and 16 GB of RAM.

## 3. Results

This study aimed to predict the binary level of nicotine dependence, defined on the basis of the Fagerström Test for Nicotine Dependence (FTND), using complete blood count (CBC) parameters, demographic and anthropometric variables, and duration of smoking exposure (years). In the dataset comprising a total of 660 patients, participants were divided into two groups according to their FTND score: patients with FTND < 6 were assigned to the Low dependence group (237 patients; 35.9%) and patients with FTND ≥ 6 to the High dependence group (423 patients; 64.1%). Accordingly, the naive baseline performance based on the majority class is 64.1%. The dataset was split into training (528 patients) and test (132 patients) subsets in an 80:20 ratio. Because one of the six FTND items directly scores the number of cigarettes smoked per day, packs per day and the pack × year variable derived from it are components of the target. In order to prevent target leakage, both were excluded a priori from the candidate feature pool, and smoking duration, which is not an FTND item, was retained as the sole predictor of smoking exposure.

During the analysis, a total of 49 candidate features were generated from the original variables through feature engineering; these were grouped as demographic/anthropometric (5), smoking (1: smoking duration), raw CBC (17), derived ratios/indices (6: BMI, BMI category, NLR, PLR, MLR, SII) and interaction terms (20). No imputation was applied, and the ratios were computed directly. From this pool of 49 features, 13 features were selected by Recursive Feature Elimination (RFE). Seven different machine learning models were used for classification: Logistic Regression, Random Forest, Gradient Boosting, SVM (RBF kernel), LightGBM, XGBoost and a stacking (STACKING) ensemble. In order to increase the reliability of the models, leakage-free 10-fold cross-validation was applied, in which feature selection and scaling were repeated within each fold; in addition, ROC-AUC values were computed on the hold-out test set. Model outputs were evaluated both at the default decision threshold (0.5) and at the optimum threshold determined by the Youden index. Model performances are presented in Table 2.

### 3.1. Model Performances

The accuracy values of all seven models remained above the baseline performance level (64.1%), but this difference was limited (0.667–0.697). According to the ROC-AUC values on the hold-out test set, the highest performance was achieved by the STACKING ensemble (Test AUC = 0.749), followed by Logistic Regression (0.746), Random Forest (0.724) and XGBoost (0.719). In contrast, according to the leakage-free 10-fold cross-validation results, which eliminate optimism bias, the highest performance was observed at an equal level in the SVM (RBF) and Logistic Regression models (CV-AUC = 0.652 ± 0.078 and 0.652 ± 0.090, respectively; mean ± SD of the ten fold-wise AUCs), followed by Random Forest and STACKING at 0.638. The difference between these two metrics is notable: while STACKING showed the highest discriminative power on the test set, no model exceeded the level of 0.66 in terms of generalizability across folds. Repeating the leakage-free 10-fold cross-validation with ten different random partitions gave mean CV-AUCs of 0.653 for logistic regression and 0.655 for SVM (SD of the ten repetition means 0.006 and 0.008), within 0.003 of the single-run estimates, indicating that the cross-validated estimates are stable. In a sensitivity analysis in which RFE and scaling were re-fitted within each inner fold of the stacking ensemble, the ensemble’s cross-validated AUC was 0.629 ± 0.078 (versus 0.638 ± 0.078) and its hold-out test AUC 0.753 (versus 0.749).

As shown in Table 2, at the default threshold of 0.5 the highest sensitivity values were obtained with the SVM (0.918) and the Random Forest and Gradient Boosting models (both 0.812), whereas these models exhibited relatively low specificity values (0.255, 0.426 and 0.404, respectively).

Logistic Regression, in turn, presented the most balanced profile between sensitivity (0.706) and specificity (0.681). At the default threshold, the highest accuracy was obtained with the Logistic Regression model (0.697), followed by STACKING (0.689) (see Table 2).

### 3.2. Decision Threshold Optimization

In order to assess the effect of class imbalance (64.1% to 35.9%) on the decision threshold, the optimum threshold maximizing the Youden index (sensitivity + specificity − 1) was determined for each model on the training data by cross-validation and applied unchanged to the hold-out test set (Table 3).

When the Youden threshold was applied in the study, the sensitivity-specificity balance of the models changed. For example, when the threshold for Logistic Regression was moved to 0.482, sensitivity rose from 0.706 to 0.741, while specificity declined from 0.681 to 0.660. With the optimum threshold (0.409), the STACKING model achieved the highest F1 score among all models (0.798) and, together with Logistic Regression, the highest accuracy (0.712). In the XGBoost model, the low threshold value (0.389) raised sensitivity to 0.882 while reducing specificity to 0.277; this shows that the choice of threshold should be adjusted according to clinical priority (the balance between not missing high dependence and limiting false positives).

**Figure 2:**
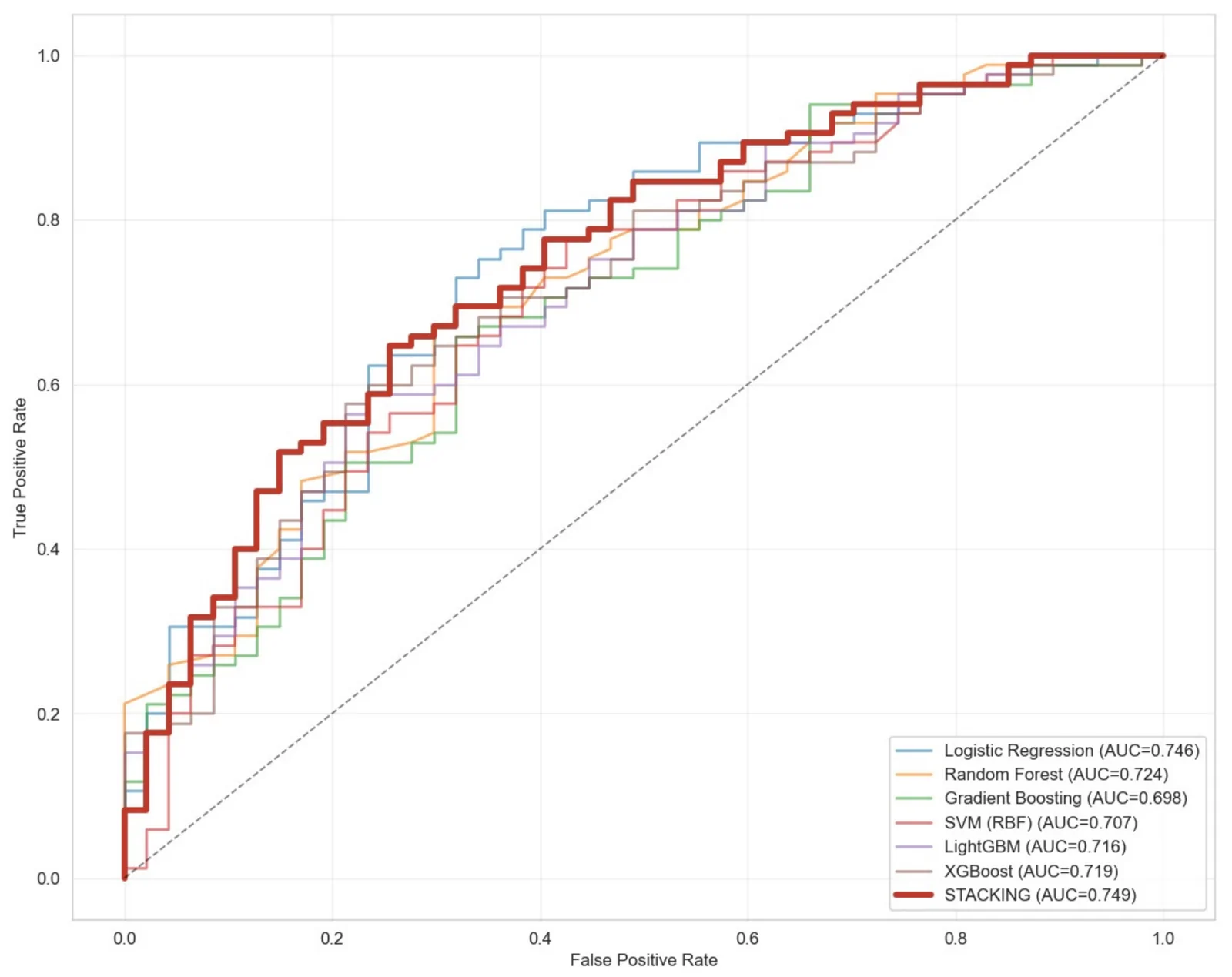
ROC curves and AUC values of the seven models on the hold-out test set. The dashed diagonal line represents a random classifier (AUC = 0.50).

The ROC curves of the seven models on the hold-out test set are presented in Figure 2. All models lie above the diagonal line representing a random classifier. The largest area under the curve belongs to the STACKING model (AUC = 0.749); Logistic Regression (0.746), Random Forest (0.724), XGBoost (0.719) and LightGBM (0.716) showed values close to one another, whereas SVM (0.707) and Gradient Boosting (0.698) had the lowest AUC values. No model reached the threshold of 0.80.

**Figure 3:**
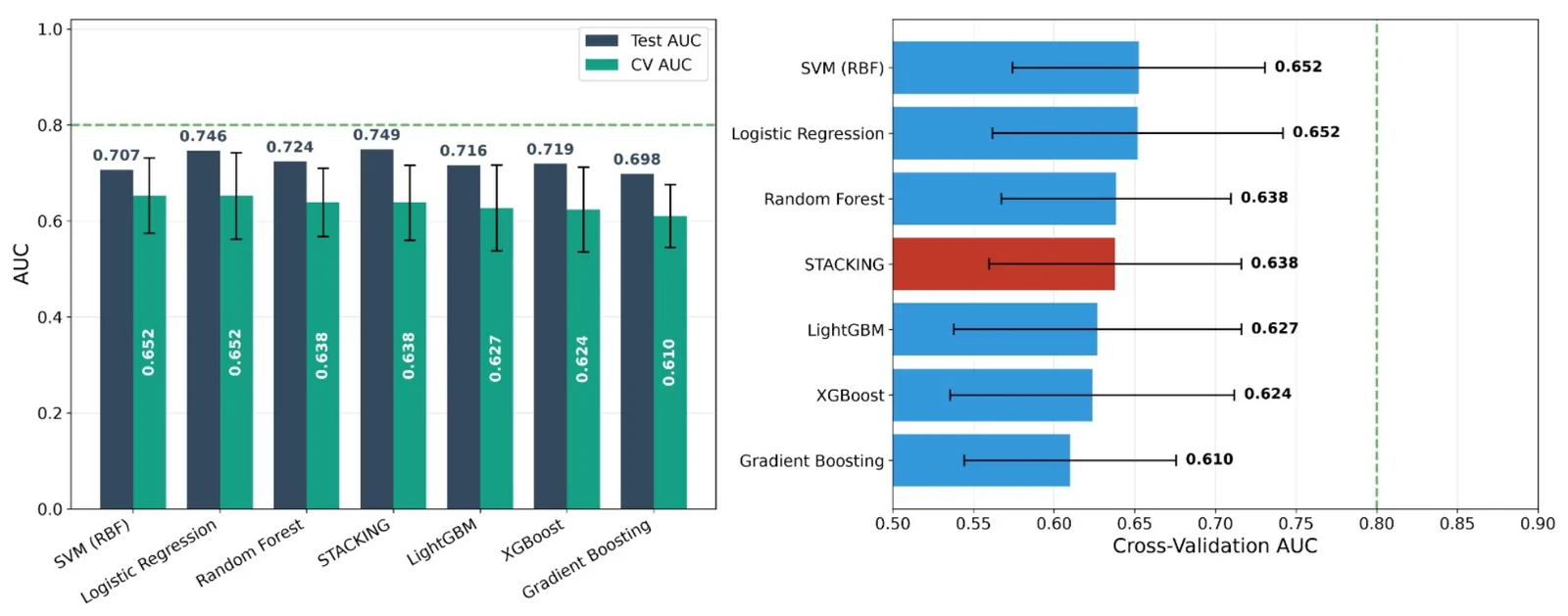
Comparison of the test set AUC values of the models with their leakage-free 10-fold cross-validation AUC values. Error bars show ±1 standard deviation across the ten folds; the dashed line marks the AUC threshold of 0.80, and the stacking ensemble is shown in red.

A comparison of the test set performance of the models with their leakage-free cross-validation performance is given in Figure 3. In all models, the test AUC values are higher than the cross-validation AUC values. This difference is compatible with sampling variability in the small hold-out test set (*n* = 132; the 95% confidence intervals of the test AUCs are wide and overlap with the cross-validated intervals, Table 2) and with the slightly smaller training partitions used in cross-validation (475–476 versus 528 participants); an optimistic single-split estimate may also contribute, but the difference alone does not demonstrate it. The leakage-free 10-fold cross-validation, which averages over ten validation folds, therefore provides the more stable estimate. In the cross-validation AUC ranking, SVM (RBF) (0.652 ± 0.078) and Logistic Regression (0.652 ± 0.090) share first place at an equal level; however, the error bars overlap widely across the models, indicating that the differences between the models are not statistically pronounced.

On the hold-out test set (Supplementary Figure S1), the Brier scores of the seven models ranged from 0.198 (Random Forest) to 0.225 (Gradient Boosting), compared with 0.229 for the non-informative reference model (Logistic Regression 0.207, SVM 0.201, STACKING 0.209). The calibration slopes of Logistic Regression, Random Forest, SVM and STACKING did not differ significantly from 1 (1.18–1.73; all 95% CIs including 1), whereas the three boosting models produced overly extreme probabilities (slopes 0.39–0.56, with upper 95% confidence limits below 1). Logistic Regression and STACKING systematically underestimated the probability of high dependence: their mean predicted probabilities were 0.53 and 0.52, whereas the observed proportion was 0.64 (calibration intercepts 0.53 [95% CI 0.16–0.90] and 0.58 [0.20–0.95]). For Logistic Regression, the mean predicted probability rose from 0.34 in the lowest to 0.74 in the highest quintile of predicted probability, and the observed proportion rose from 0.33 to 0.92; in four of the five quintiles, the observed proportion exceeded the mean prediction. This shift is an expected consequence of the balanced class weighting of the logistic model that produces the final probabilities (the model itself for Logistic Regression and the meta-learner for STACKING); it disappeared when the predicted log-odds were shifted by ln(338/190), the logarithm of the training-set class ratio (calibration intercepts −0.05 and 0.00; Brier scores 0.194 and 0.193), a correction that does not change discrimination. The SVM, whose probabilities are obtained by internal Platt scaling, was well calibrated in the large (intercept −0.01).

The pooled out-of-fold predictions of the leakage-free cross-validation (*n* = 528) showed somewhat poorer calibration. The Brier scores ranged from 0.212 (SVM) to 0.256 (Gradient Boosting), against a reference value of 0.230. Except for Gradient Boosting (not class-weighted) and the Platt-scaled SVM, all models underestimated the probability of high dependence (calibration intercepts 0.23–0.57), most markedly Logistic Regression (Brier score 0.235) and STACKING (0.231); after the class-ratio correction, the intercepts of these two models were −0.01 and −0.02 and their Brier scores were 0.216 and 0.215. The calibration slopes were below 1 for most models (0.24–0.32 for the boosting models, 0.68 for Random Forest and 0.72 for Logistic Regression), indicating overfitting, and the SVM was the best-calibrated model (intercept −0.01, slope 0.95). Thus, although the models rank patients better than chance, their predicted probabilities should not be interpreted as estimates of an individual patient’s probability of high dependence without recalibration.

### 3.3. Feature Selection and Ablation Analysis

The 13 features selected by RFE from the pool of 49 candidate features, together with their types, are listed in Table 4. Eleven of the selected features are products of feature engineering (1 derived index: BMI; 10 interaction terms), whereas 2 are raw variables (smoking duration and platelet count). This distribution shows that the data-driven selection preferentially retained the engineered features. Because these features are correlated with their components and with one another (see below), the information they carry largely overlaps with that of the raw variables, which is consistent with the ablation analysis (Table 5).

As expected for engineered features, the selected features were intercorrelated. The product terms, which were specified a priori on domain grounds (Section 2.5), were correlated with their component variables (r = 0.27–0.98) and with one another; overall, 14 of the 78 feature pairs had |r| > 0.7 (maximum r = 0.96), and 9 of the 13 features had a VIF above 10 (range 1.2–130.5; Table 4). Such multicollinearity does not reduce predictive performance, which is captured by the leakage-free cross-validated estimates, but it affects how the contribution is distributed among correlated features (Section 3.4). In univariate analysis, all 13 selected features were positively associated with the FTND score (Spearman ρ = 0.09–0.37; Table 4).

**Figure 4:**
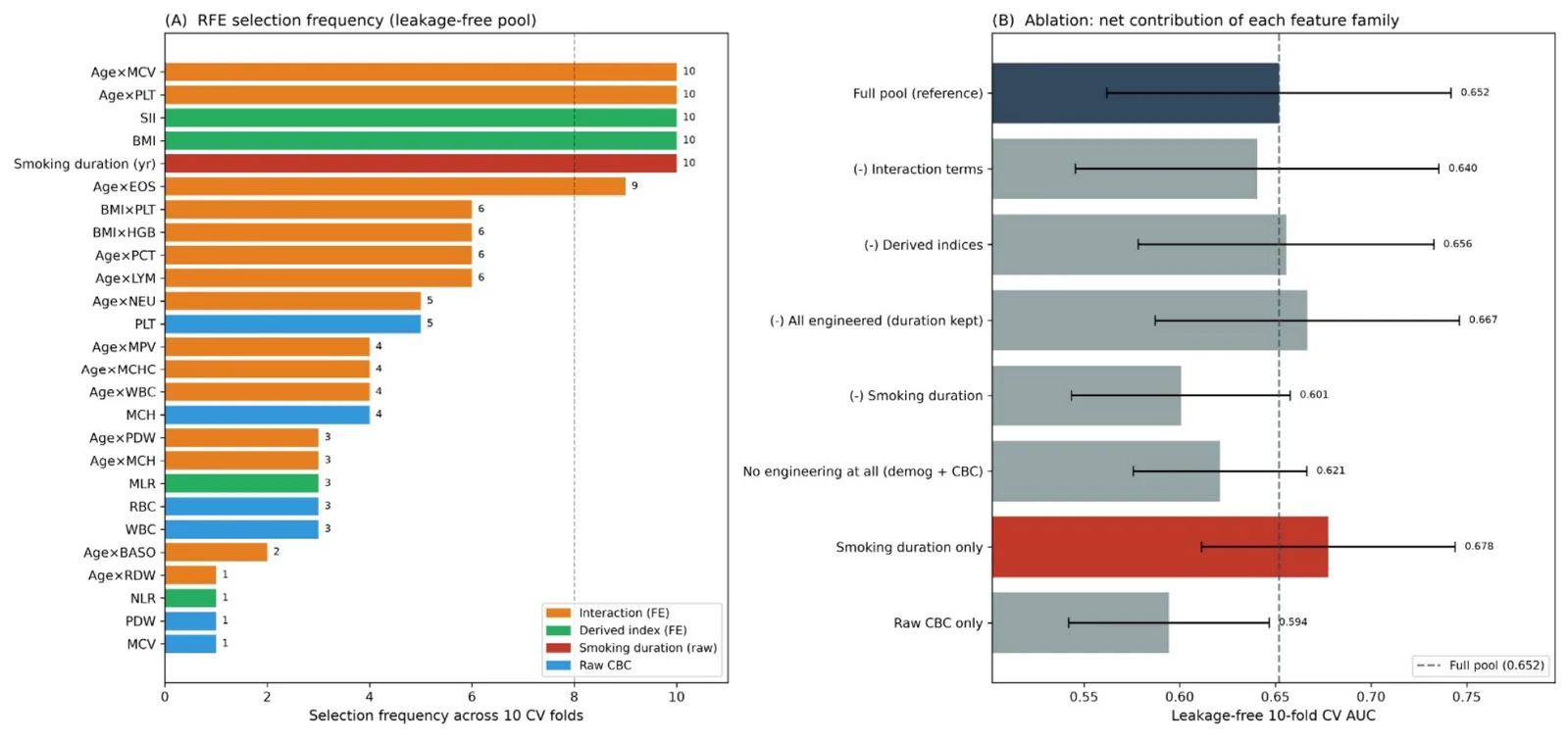
(**A**) RFE feature selection frequency across the 10 cross-validation folds; the dashed line marks the threshold for stable selection (at least 8 of the 10 folds). (**B**) Ablation analysis of the net contribution of the feature groups (leakage-free 10-fold CV AUC); the full feature pool (reference) is shown in dark blue, smoking duration only in red and the other feature sets in gray. Error bars show ±1 standard deviation across the ten folds.

The stability of feature selection across folds is shown in Figure 4A. Five features, smoking duration, BMI, SII, age × platelets and age × mean corpuscular volume, were selected in all 10 folds (selection frequency = 10), followed by age × eosinophils (9) and by BMI × platelets, BMI × hemoglobin, age × plateletcrit and age × lymphocytes (6 each). While the final selected set (Table 4) is based on the selection made on the training set, Figure 4A reflects the consistency of selection across folds and reveals that duration of smoking exposure together with the platelet- and inflammation-based features carry the most stable discriminative information.

**Figure 5:**
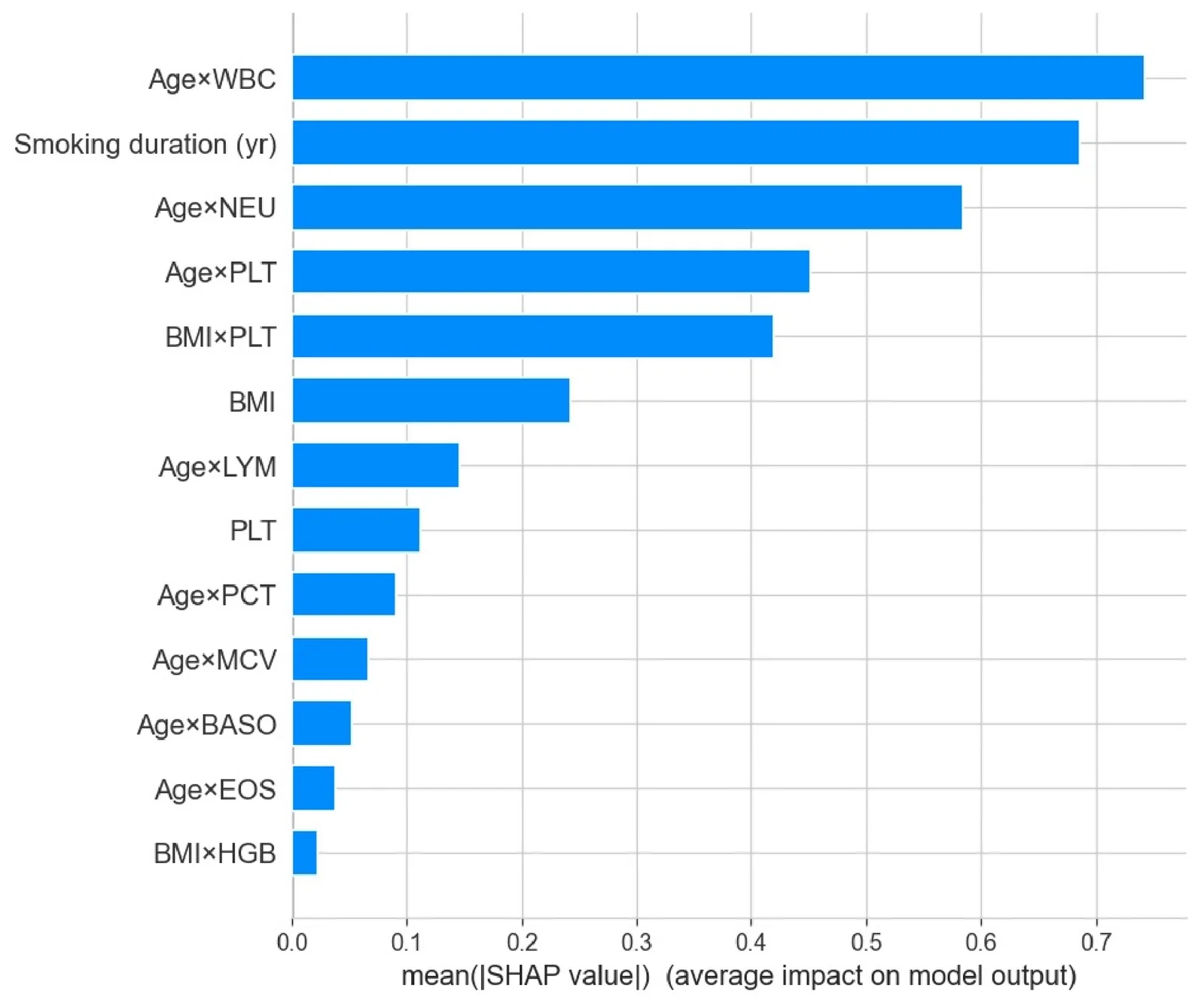
Bar chart of the SHAP global feature importance of the 13 selected features (mean |SHAP| value; logistic regression model, training set, *n* = 528). The numerical values are listed in Table 8.

In order to quantify the contribution of the feature groups to the model, an ablation analysis was performed, and the results are presented in Figure 4B and Figure 5 (leakage-free 10-fold CV AUC). The full feature pool gave a reference AUC value of 0.652. Removing the interaction terms (0.640) or the derived indices (0.656) did not change performance meaningfully; indeed, when all feature engineering was removed and only the raw and demographic variables were used, the AUC rose slightly (0.667). In contrast, removing smoking duration lowered performance from 0.652 to 0.601. When smoking duration was used alone, an AUC of 0.678 was obtained, a value above that of the full 49-feature pool, whereas the 17 raw CBC parameters alone gave an AUC of only 0.594. These findings show that the discriminative power of the model derives almost entirely from the duration of smoking exposure, that the raw CBC parameters alone provide discrimination close to chance level, and that extensive feature engineering provided no measurable gain.

The differences between the three main configurations were then tested formally on the pooled out-of-fold predictions (Table 6). The full 49-feature model did not perform better than smoking duration alone: its pooled cross-validated AUC was lower (ΔAUC = −0.021; bootstrap 95% CI −0.052 to +0.012), and the difference was not statistically significant (DeLong *p* = 0.22). In contrast, both the full model and smoking duration alone discriminated significantly better than the 17 raw CBC parameters (full model vs. raw CBC: ΔAUC = +0.063, DeLong *p* = 0.016; smoking duration vs. raw CBC: ΔAUC = +0.083, DeLong *p* = 0.010). The additional CBC and engineered features therefore provided no statistically detectable improvement over smoking duration alone, and the discriminative signal resided in the duration of smoking exposure rather than in the hematological features.

To assess whether the findings depend on the chosen cut-off, the leakage-free evaluation was repeated at two additional clinically used cut-offs bracketing the primary threshold, FTND ≥ 5 and FTND ≥ 7 (Table 7). The main findings were robust to the cut-off: at every threshold, the full 49-feature model provided no meaningful gain over smoking duration alone (0.732 vs. 0.726 at ≥5; lower at ≥6 and ≥7), and the raw CBC panel remained clearly the weakest. The absolute AUC varied with the cut-off, being higher at ≥5 and lower at ≥7, as expected from the change in class balance and task difficulty, whereas the relative pattern (smoking duration ≈ full model ≫ raw CBC) was unchanged; the results therefore do not depend on a particular threshold. When the FTND score was modeled as a continuous outcome, the relative performance of the feature configurations was unchanged, the full model performed similarly to smoking duration alone, and the raw CBC panel was the weakest, and the continuous predictions discriminated FTND ≥ 6 as well as the binary logistic regression models (AUC of the pooled out-of-fold predictions of the linear regression 0.669 for the full model, 0.676 for smoking duration alone and 0.589 for the raw CBC panel, compared with 0.654, 0.674 and 0.591 for the binary models), with the same pattern for the random-forest regressor. In cross-validation, the linear regression of the full pool predicted the FTND score with a mean absolute error (MAE) of 1.96 points, a root mean squared error (RMSE) of 2.40 and an R^2^ of 0.12 (random-forest regression: 1.91, 2.37 and 0.15), compared with 2.08, 2.56 and 0.00 for a model predicting the training mean; smoking duration alone gave similar values (1.98, 2.41 and 0.11), whereas the raw CBC panel explained almost none of the variance (R^2^ = 0.03).

When the leakage-free 10-fold cross-validation was repeated for 1 to 49 features (odd values), performance was largely insensitive to the number of features: for 5 to 49 features, the CV-AUC ranged from 0.641 to 0.671 for logistic regression and from 0.625 to 0.664 for SVM, and between 11 and 17 features (the upper end of the range compatible with at least 10 events per variable) it varied by no more than 0.02 (logistic regression 0.646–0.652; SVM 0.634–0.652). With one or three features, the CV-AUC was lower (0.611–0.625); in all ten folds, the top-ranked single feature was an age- or BMI-based interaction term, and smoking duration was not among the top three features. Smoking duration was among the top five RFE-ranked features in nine of the ten folds and among the 13 selected features in all ten.

### 3.4. Explainable Artificial Intelligence: SHAP and LIME

SHAP and LIME methods were applied in order to interpret the model’s predictions. SHAP values were computed with LinearExplainer on the Logistic Regression model. The global feature importance ranking (mean |SHAP| value) is given in Table 8 and Figure 5. The greatest contribution was made by the age × leukocyte interaction (mean |SHAP| = 0.742), followed by smoking duration (0.686), age × neutrophils (0.584), age × platelets (0.451) and BMI × platelets (0.419). With the outcome-overlapping consumption variables excluded a priori, no single feature dominated, and the contribution was distributed across several features.

**Figure 6:**
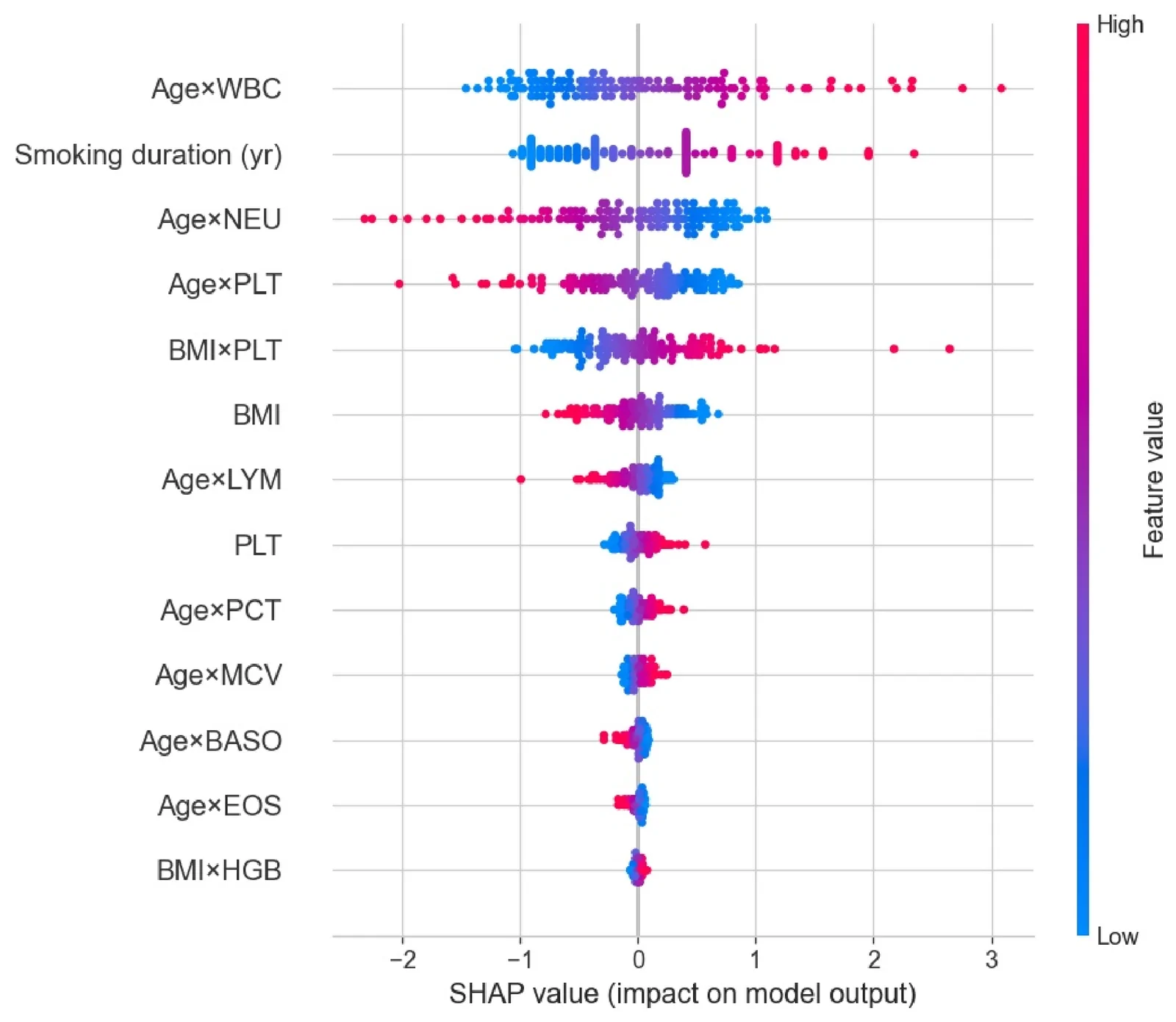
SHAP beeswarm plot: the magnitude and direction of the features’ contributions to the model output at the individual patient level (logistic regression model; training set, *n* = 528).

Because of this multicollinearity, the SHAP values of individual features should be interpreted with caution. Correlated features can carry weights of opposite sign that partly offset one another: in the logistic regression model, six of the 13 features carried negative weights despite their positive univariate associations (Figure 6). For example, age × leukocytes and age × neutrophils (r = 0.95) had individual mean |SHAP| values of 0.742 and 0.584, whereas their combined contribution was 0.243. The SHAP values therefore describe predictive contributions within this model and do not provide evidence of biological interaction or of independent effects.

The SHAP beeswarm plot (Figure 6) shows the magnitude and direction of each feature’s contribution to the model output at the individual patient level. For smoking duration, high values (red) are associated with positive SHAP values, that is, with a prediction toward High dependence, whereas low values (blue) push the model toward Low dependence. This pattern is consistent with the expected positive association between smoking duration and high dependence.

For comparison, the intrinsic feature importance values of the Random Forest model were also examined (Supplementary Figure S2). In this model, smoking duration emerged as the feature with the highest importance value (importance ≈ 0.105), followed by BMI (≈0.082) and age × platelets (≈0.079). However, the differences between the features are small; the remaining features cluster at close values in the range 0.066–0.079. Consistent with the SHAP analysis, this pattern confirms that the discriminative information is not concentrated in a single feature.

For local (patient-level) explanations, the LIME method was applied to two example patients with the highest and the lowest predicted probability. Supplementary Figure S3 shows a patient for whom the probability of High dependence was predicted as *p* = 0.875 (true class: High). In this patient, the largest contributions toward a High prediction came from age × leukocytes (+0.287) and smoking duration (+0.247), whereas age × neutrophils (−0.232) and age × platelets (−0.203) contributed in the opposite direction. As described above, these opposite-signed contributions reflect the multicollinearity among the selected features rather than opposite biological effects.

Supplementary Figure S4, in turn, shows a patient for whom the probability of High dependence was predicted as *p* = 0.208 (true class: Low). Here the largest contributions came from the low values of age × leukocytes (−0.213) and smoking duration (−0.187), which moved the prediction toward Low. Consistent with the global SHAP findings, both LIME examples show that smoking duration and the age-based interaction terms make the largest contributions to individual predictions.

In our study, measurements were taken as a single observation per patient, feature selection was repeated within each fold, and variables that are components of the outcome were excluded before the analysis. These choices lowered the reported performance but ensured that the value obtained reflects the true information content of routine clinical data.

Both the ablation and the explainability analyses (SHAP, LIME and random forest feature importance) show that the discriminative power of the model derives predominantly from the duration of smoking exposure, and that routine complete blood count parameters alone provide discrimination close to chance level (AUC = 0.594). Considered as a whole, all models performed only slightly above the baseline performance of 64.1%; the highest AUC value on the hold-out test set was obtained with the STACKING ensemble (0.749), and the highest AUC value in the leakage-free cross-validation with SVM (RBF) and Logistic Regression (both 0.652). This level is consistent with the performances reported with multivariable machine learning models in the field of addiction.

### 3.5. Association of the Variables with the Level of Dependence

Independently of the machine learning models, the univariate association of the examined variables with the Fagerström dependence score was assessed with Spearman rank correlation, and differences between the High and Low dependence groups were compared with the Mann–Whitney U test (see ‘Statistical analysis’). The FDR-corrected results are summarized in Table 9. Packs per day showed the strongest correlation with the level of dependence (ρ = +0.551); this is expected by construction, because the number of cigarettes smoked per day is one of the six FTND items, and packs per day is therefore reported in Table 9 only as an outcome-overlapping reference variable, not as independent evidence. Among the variables that are not part of the FTND, the strongest associations were shown by smoking duration (ρ = +0.353) and age (ρ = +0.247). Because smoking duration was strongly correlated with age (r = 0.83), the two were also examined jointly. After mutual adjustment, smoking duration remained associated with the FTND score (partial ρ = +0.27, *p* < 0.001), whereas age was no longer positively associated with it (partial ρ = −0.08, *p* = 0.03). Consistently, in the leakage-free cross-validation, age alone discriminated less well than smoking duration alone (mean fold-wise CV-AUC 0.611 versus 0.678; DeLong test on the pooled out-of-fold predictions, *p* < 0.001), and adding age to smoking duration did not improve discrimination (0.682; *p* = 0.31).

Some of the raw complete blood count parameters showed statistically significant but, in terms of magnitude, weak associations; even for the leukocyte count, the strongest hematological variable, the correlation remained at ρ = +0.184 (FDR *p* < 0.001). Lymphocytes, neutrophils, red cell distribution width, platelets, eosinophils and plateletcrit likewise showed weak but significant positive associations (all ρ values < 0.19). This finding shows that the large sample (*n* = 660) can render even small effect sizes statistically significant, while these associations remain clinically limited.

Notably, none of the four indices derived as indicators of chronic inflammation (NLR, PLR, MLR and SII) showed a significant association with the Fagerström score (FDR-corrected *p* > 0.10 for all). Whereas neutrophil, lymphocyte and platelet parameters, the components of these indices, were individually associated with dependence in a weak but positive manner, the loss of this association in the indices obtained by taking their ratios suggests that, in this dataset, the ratio transformation masks the existing weak signal rather than providing additional information. This observation is consistent with the unstable behavior of the same indices in feature selection and with their limited contribution as detected in the ablation analysis, and it supports, through an independent method, the general finding that the routine hemogram carries a real but weak signal regarding dependence severity.

## 4. Discussion

In this study, the classifiability of nicotine dependence severity as defined by the FTND was investigated using routine complete blood count (CBC) parameters, demographic/anthropometric variables and smoking duration. The main element distinguishing the design of this study from comparable ones is the a priori exclusion from the candidate feature pool of the variables that are components of the outcome, packs per day and the pack × year variable derived from it, on the grounds of target leakage [17]. This choice lowered the apparent performance but made it possible to obtain a bias-free estimate of the extent to which routine clinical data can in fact discriminate dependence severity. The findings are discussed below around this axis.

### 4.1. Target Leakage and the Source of Apparent Performance

The FTND is a six-item self-report scale [2,3], and one of its items directly scores the number of cigarettes smoked per day, contributing up to 3 of the total 10 points. Consequently, the amount of daily consumption is not an independent predictor of the level of dependence but a component of the outcome definition itself. When an FTND-based classification is performed using the pack × year variable derived from it, the model partly predicts the outcome from itself; this is a problem defined in the data mining literature as target leakage, which artificially inflates predictive performance [17].

The ablation analysis of our study made it possible to measure quantitatively where the source of the apparent performance lies in this dataset (Table 5). In the 49-variable pool, from which the consumption variables overlapping with the outcome were excluded a priori, the leakage-free 10-fold cross-validation AUC value is 0.652. When smoking duration alone is removed from this pool, the AUC falls to 0.601; in contrast, when smoking duration is used alone, it reaches 0.678, a value above the performance of the full 49-variable pool. The raw complete blood count parameters alone, on the other hand, give an AUC of only 0.594. Taken together, these three observations show that, even after the variables overlapping with the outcome have been excluded, the discriminative power of the model derives largely from smoking duration, the legitimate measure of exposure, whereas routine hemogram patterns alone make only a limited contribution. The full multivariable model is therefore not proposed as a predictor in its own right; its role in this study is analytical. As the reference model of the ablation analysis, it shows that routine hemogram parameters, demographic variables and engineered features add no measurable discrimination to smoking duration, a single measure of exposure that is available from the history at no cost (Section 3.3). On grounds of parsimony, smoking duration alone would be the preferable predictor, although its own discrimination (AUC = 0.678) is also insufficient for classification at the individual level.

The dominance of smoking duration raises the question of what it represents. Because its association with dependence persisted after adjustment for age, whereas that of age did not (Section 3.5), smoking duration reflects cumulative exposure, at a given age, an earlier start of smoking, rather than age itself. The model therefore predicts dependence as defined by the FTND, not exposure; the exposure-intensity item of the scale (cigarettes per day) was excluded from the predictors, but it captures mainly the part of dependence that covaries with cumulative exposure, whereas components of dependence that are independent of exposure are not captured by routine clinical data.

For this reason, the consumption variables were excluded a priori in our study, and the final model was built on a pool of 49 variables. The cost of this choice is a marked decrease in the reported performance; its benefit is that the value obtained reflects the true information content of routine clinical data. In our view, this is not merely a methodological detail but one of the principal contributions of the study: in designs where FTND-based outcomes are predicted with consumption amount variables, it is often not disentangled how much of the reported performance derives from a genuine biological signal and how much from overlap by definition. The ablation we present constitutes a concrete example of how this distinction can be made on the same data.

### 4.2. The True Discriminative Power of the Routine Hemogram

The performance obtained when the leaky variables are excluded is above chance level (AUC = 0.50) but moderate. In the scenario in which the raw CBC parameters were used alone, the AUC remained at 0.594; with the addition of the demographic variables, this value rose to 0.621 (Table 5). Formal comparison showed that the full model did not discriminate significantly better than smoking duration alone (DeLong *p* = 0.22) and that its advantage over the raw CBC panel derived from the duration of smoking exposure rather than from the hematological features (Table 6). This picture shows that the routine hemogram carries a real but weak signal regarding dependence severity.

This pattern was consistent across all seven classifiers (Table 2): no model exceeded a leakage-free cross-validated AUC of 0.66, and at the default threshold accuracy remained only slightly above the naive baseline of 64.1%.

This result is biologically consistent with three mutually independent lines of evidence. First, although the effect of smoking on blood cell indices has been demonstrated causally, the effect size is small. Pedersen et al., in a Mendelian randomization study covering 104,607 individuals, reported that smoking causally increased leukocyte, neutrophil, lymphocyte and monocyte counts as well as hematocrit, hemoglobin and mean corpuscular volume, but that in the genetic analyses the percentage differences per T allele remained at the level of 1.15% for leukocytes, 1.07% for neutrophils, 1.34% for lymphocytes and 1.50% for monocytes [30]. Compared with the natural inter-individual variability, differences of this magnitude cannot be expected to provide strong discrimination at the individual level.

Second, clinical studies examining the association of hemogram-based indicators with the level of dependence report differences that are statistically significant but whose distributions overlap widely. Güden et al. showed, in 247 chronic smokers, that as the level of dependence increased the leukocyte count rose from 7.8 ± 1.4 to 8.8 ± 1.4, and that NLR and PLR were significantly associated with the level of dependence, exhaled carbon monoxide and the amount of exposure [13]. Similarly, the effects of smoking on NLR, PLR and platelet indices have also been reported in different samples [10,11]. However, these findings are at the level of group means; the overlap of the standard deviations prevents the same associations from translating into strong classification at the individual patient level. At this point our study advances the literature and documents quantitatively the rate at which associations demonstrated at the group level translate into individual prediction.

Third, and conceptually the most decisive, hemogram changes reflect not dependence itself but exposure and the systemic inflammation associated with it. Indeed, these indicators have been shown to improve markedly after smoking cessation [12] and to be associated with exhaled carbon monoxide levels; the changes observed in NLR, PLR and related ratios after smoking cessation are in the same direction [31,32]. Dependence, on the other hand, is a behavioral and neurobiological construct, and its relationship with the hemogram is therefore likely to be indirect, through exposure; the present cross-sectional design cannot, however, test such mediation. For this reason, when the direct measure of exposure is removed from the pool, only an indirect and attenuated signal would be expected to remain. Our findings are consistent with this interpretation, and the moderate performance observed may reflect the nature of this relationship rather than a shortcoming of the model.

### 4.3. Task Difficulty and Comparison with the Literature

In order for the performance obtained to be interpreted, the level of difficulty of the problem being solved must be taken into account. Distinguishing smokers from never-smokers is structurally a much easier problem than grading dependence severity among smokers; in the former case there is a qualitative boundary between the groups, such as the presence/absence of exposure, whereas in the latter all individuals have been exposed and the difference is only one of degree.

Quantitative evidence for this distinction is seen in the study by Reinhardt et al. In a population-based cohort of 707 smokers and 864 never-smokers, using EEG together with sociodemographic and environmental data and applying nested cross-validation repeated 10 times, the authors obtained an AUROC of 0.85 ± 0.03 between smokers and never-smokers and of 0.92 ± 0.03 between heavy smokers and never-smokers. In contrast, in the same cohort, performance in the quitter/non-quitter classification, which requires a within-group distinction, fell to 0.68 ± 0.13, and the EEG domain alone remained at chance level with 0.51 ± 0.17 [33]. This picture shows how difficult it is, even with expensive and multimodal data, to disentangle the subtle differences within a tobacco-using population.

The objective of our study also belongs to this class of difficulty. It is noteworthy that the value obtained in our leakage-free design is of the same order of magnitude as the within-group performance of the aforementioned study, which used multimodal and costly data; in the interest of candor, however, it must be stated that our value is somewhat below the level of 0.68 reported by that study. On the other hand, the comparison should be made taking into account the cost and accessibility of the data source used: while the model of Reinhardt et al. requires EEG recording and detailed sociodemographic and environmental assessment, our model relies only on a blood test that is already requested at every clinical presentation and on a few basic demographic variables. Moreover, the two tasks are not identical; the study in question predicts cessation success, whereas our model predicts dependence severity.

In contrast, studies targeting the binary smoker/non-smoker distinction report markedly higher values, as would be expected. Aishwarya et al. obtained an accuracy of 0.80 and an AUC of 0.84 in an artificially balanced clinical dataset, and studies applying deep learning to structural MRI have likewise distinguished smokers from non-smokers with high accuracy [34]. The fact that these values are above our result stems from the difference in task definition rather than from model quality, and a direct comparison would be misleading.

The few studies that attempt to predict the level of dependence from variables that do not overlap with the outcome support the difficulty we encountered. Kharabsheh et al. built a three-class model in waterpipe smokers with demographic features such as age, place of residence and education; although they obtained a sensitivity of 0.82, the precision value remained at 0.43 [16]. This example shows how performance is constrained when working with variables that are not components of the outcome. Başçıl and İşcanlı, who inferred the level of dependence from physiological signals, reported high accuracy in a four-class formulation [9]. In interpreting such high values, it would be useful to take into account the possible effects of measurement timing and group definitions on the result. In general, we are of the opinion that in comparing performance values in this field one should consider not only the numerical magnitude but also the relationship between the outcome definition and the feature pool.

### 4.4. Methodological Appraisal

The principal elements that determine the methodological position of our study are summarized below.

First is the exclusion, prior to the analysis and on theoretical grounds, of the variables that are components of the outcome. Such an exclusion was made not because it lowered performance after the model had been built, but because it is conceptually necessary when the outcome definition is examined; moreover, the quantitative consequence of this decision was reported transparently by means of the ablation analysis.

Second is the leakage-free nature of the evaluation protocol. Feature selection and scaling were re-performed only on the training portion of each cross-validation fold, and the validation portion was never accessed at any stage. This design eliminates the optimism bias arising from performing feature selection once on the entire dataset. For comparison, some studies in the relevant literature report their performance on a single training/test split.

Third is ablation-based transparency. The net contribution of the feature groups was measured separately, so that both the contribution of smoking duration and the limited gain from feature engineering in this dataset could be reported explicitly. We are of the opinion that a design that reports the limits of its own methodological choices in this way increases the reliability of the results.

Fourth is the two-level use of explainability. By producing global explanations with SHAP and patient-level local explanations with LIME, the variables on which the model relies were made transparent. These explanations show which routine parameters the model uses for its predictions; they describe predictive contributions and do not by themselves establish a biological or causal relationship with dependence severity. The growing emphasis on interpretability in AI-based medical prediction is also illustrated by recent image-based oncology frameworks that combine diagnostic classification with prognostic prediction or explainable interpretation [35,36].

Fifth is the use of a dataset that comes from real clinical conditions and is naturally imbalanced. Rather than being resolved through artificial sampling, class imbalance was addressed through class weighting (Section 2.7), and the effect of the decision threshold was examined separately (Section 3.2), with the aim of keeping the reported performance closer to the conditions of clinical practice.

Tomko et al., in a clinical research sample of 302 patients, reported AUC values between 0.723 and 0.773 and accuracy between 0.665 and 0.728 in 10-fold cross-validation for models built with 70 baseline variables spanning the demographic, medical, psychiatric and substance use domains; they explicitly stated that the best model, with 73% accuracy, could not be used on its own and that performance would need to be improved further for clinical decisions. The same study summarizes the classification rates of machine learning studies predicting substance use treatment response as 50–82% and their AUC values as being in the range 0.67–0.89 [37]. Our test set AUC value (0.749) falls within this range, whereas our leakage-free cross-validation value (0.652) remains just below it. There are two reasons for this positioning: first, the outcome of our study is a within-group discrimination problem requiring severity grading among tobacco users; second, our protocol, in which feature selection and scaling are repeated only on the training portion of each fold, does not contain the optimism bias present in designs where the selection procedure is optimized directly on cross-validation performance. The difference of approximately 0.10 between the hold-out test AUC (0.749) and the leakage-free cross-validated AUC (0.652) in our own data illustrates how much a single-split estimate can differ from a cross-validated one in a dataset of this size; given the width of the test-set confidence interval (0.667–0.831), part of this difference may reflect sampling variability rather than optimistic bias alone.

### 4.5. Clinical Implications

Our findings show that routine CBC parameters do not by themselves constitute an independent diagnostic tool that can be used to determine nicotine dependence severity. The discriminative power obtained is below the level that could be considered sufficient on its own for clinical decision-making, and the explicit reporting of this result is important for establishing a realistic level of expectation in the field. In contrast, the potential of hemogram-based indicators to provide complementary information to existing assessment instruments should not be disregarded; although the inflammatory ratios showed no association with dependence severity in the present cohort (Section 3.5), the evidence that these indicators change during the smoking cessation process suggests that they may be evaluated for monitoring purposes rather than for determining severity. Rather than replacing scales such as the FTND in clinical practice, positioning them as part of a multicomponent assessment framework together with objective biochemical markers appears more realistic.

### 4.6. Limitations

This study has several limitations. First, it was a single-center, retrospective study and no external validation cohort was available; the findings therefore require confirmation in independent, multi-center data before any clinical use. Second, participants were recruited from a smoking-cessation clinic and were required to have complete data and no chronic disease or regular medication use. Although these criteria minimize confounding of the hematological parameters by comorbidities and drugs, they select a comparatively healthy subset and mean that only 660 of the 1321 screened individuals were analyzed; the results may therefore not generalize to the broader smoking population, in whom comorbidities and medications are common (selection bias and limited external validity). Third, the outcome and smoking duration were based on self-report, which introduces measurement uncertainty and places an upper bound on the achievable performance of label-based models. Fourth, the FTND score was dichotomized at the established cut-off of 6; although a sensitivity analysis at alternative cut-offs confirmed that the main findings are robust, dichotomization inevitably discards information contained in the ordinal scale. Fifth, the sample was predominantly male (68%), which may further limit generalizability. Sixth, the confidence intervals of the cross-validated AUC were derived from ten fold-wise estimates that are not independent and are therefore approximate, and the hold-out test set (*n* = 132) is small for the assessment of calibration; the confidence intervals and the calibration results should be interpreted with corresponding caution. Finally, biochemical verification of smoking status and exposure (e.g., cotinine or exhaled carbon monoxide) was not available.

## 5. Conclusions

This study assessed the extent to which nicotine dependence severity as defined by the FTND can be predicted from routine complete blood count parameters (CBC), demographic variables and smoking duration, using a leakage-free design in which the variables overlapping with the outcome were excluded a priori. Routine complete blood count parameters do not by themselves constitute an independent diagnostic tool for determining dependence severity; they carry only weak information on dependence severity, did not improve discrimination beyond smoking duration alone, and are best regarded as a possible complementary source of information rather than a screening biomarker. To our knowledge, it is among the leading studies that classify FTND-based nicotine dependence severity at the individual patient level by combining routine complete blood count parameters with machine learning and explainable artificial intelligence. The model should, however, be regarded as exploratory: it is not ready for clinical use and requires external, multi-center validation.

It is recommended that the framework set out by this study be used in future research. First, because routine complete blood count parameters are obtained at every clinical presentation without additional cost or additional procedures, they can be incorporated at negligible cost as a complementary source of information in future multivariable studies of dependence severity; however, given their weak discrimination in the present cohort (CBC-only AUC = 0.594) and the absence of a statistically detectable gain over smoking duration alone, they should not be regarded as a practical screening biomarker or as a substitute for established assessment instruments. Second, we concluded that research working with FTND-based outcomes should exclude a priori from its candidate pools the consumption amount variables that overlap with the item structure of the scale, and should report the effect of this decision transparently by means of ablation analysis; otherwise it cannot be disentangled how much of the reported performance derives from a biological signal and how much from overlap by definition. Third, it is recommended that the leakage-free evaluation protocol, in which feature selection and scaling are carried out within the cross-validation folds, be adopted in place of single-split reporting. Testing this framework in larger and multicenter cohorts, supporting the class labels with biochemical markers such as exhaled carbon monoxide or cotinine, monitoring hemogram-based indicators longitudinally throughout the cessation process, and integrating these parameters into multicomponent decision support systems together with existing assessment scales are possible directions for future research.

## Figures and Tables

**Table 1:** Composition of the 49-variable candidate feature pool used prior to feature selection. CBC, complete blood count; NLR, neutrophil-to-lymphocyte ratio; PLR, platelet-to-lymphocyte ratio; MLR, monocyte-to-lymphocyte ratio; SII, systemic immune-inflammation index.

Feature Group	Count	Variables
Demographic	5	Age, sex, height, weight, marital status
Raw CBC	17	WBC, RBC, PLT, HGB, HCT, NEU, LYM, MONO, BASO, EOS, MCH, MCHC, MCV, RDW, PCT, PDW, MPV
Derived indices	6	BMI, BMI category, NLR, PLR, MLR, SII
Interaction terms	20	Age × (each of the 17 CBC parameters); BMI × HGB; BMI × WBC; BMI × PLT
Smoking exposure	1	Smoking duration (years)
Total candidate pool	49	

**Table 2:** Performance metrics of the models on the hold-out test set (*n* = 132; 85 High, 47 Low) at the default decision threshold (0.5), together with the hold-out test AUC and the leakage-free 10-fold cross-validation AUC (training set, *n* = 528). Values in parentheses are 95% confidence intervals: Wilson score method for sensitivity, specificity and accuracy; percentile bootstrap (10,000 stratified resamples) for the F1 score; Hanley–McNeil method for the test AUC; mean ± 1.96·SD/√10 across the ten folds for the CV-AUC. SD, standard deviation.

Model	Sensitivity	Specificity	F1	Accuracy	Test AUC	CV-AUC
Logistic Regression	0.706 (0.602–0.792)	0.681 (0.538–0.796)	0.750 (0.675–0.817)	0.697 (0.614–0.769)	0.746 (0.663–0.829)	0.652 (0.596–0.708)
Random Forest	0.812 (0.716–0.881)	0.426 (0.295–0.567)	0.762 (0.705–0.817)	0.674 (0.590–0.748)	0.724 (0.638–0.810)	0.638 (0.594–0.682)
Gradient Boosting	0.812 (0.716–0.881)	0.404 (0.276–0.547)	0.758 (0.701–0.813)	0.667 (0.583–0.741)	0.698 (0.609–0.787)	0.610 (0.569–0.651)
SVM (RBF)	0.918 (0.840–0.960)	0.255 (0.153–0.395)	0.788 (0.749–0.826)	0.682 (0.598–0.755)	0.707 (0.619–0.795)	0.652 (0.604–0.700)
LightGBM	0.753 (0.652–0.832)	0.553 (0.412–0.686)	0.753 (0.683–0.814)	0.682 (0.598–0.755)	0.716 (0.629–0.803)	0.627 (0.572–0.682)
XGBoost	0.753 (0.652–0.832)	0.511 (0.372–0.647)	0.744 (0.675–0.805)	0.667 (0.583–0.741)	0.719 (0.632–0.806)	0.624 (0.569–0.679)
STACKING	0.741 (0.639–0.822)	0.596 (0.453–0.724)	0.754 (0.683–0.818)	0.689 (0.606–0.762)	0.749 (0.667–0.831)	0.638 (0.590–0.686)

**Table 3:** Model performance metrics on the hold-out test set at the optimum decision threshold determined by the Youden index. For each model, the threshold was selected on the training data by 10-fold cross-validation (out-of-fold predictions) and applied unchanged to the hold-out test set. Values in parentheses are 95% confidence intervals (Wilson score method; F1 score: percentile bootstrap with 10,000 stratified resamples).

Model	Youden Threshold	Sensitivity	Specificity	F1	Accuracy
Logistic Regression	0.482	0.741 (0.639–0.822)	0.660 (0.517–0.778)	0.768 (0.697–0.831)	0.712 (0.630–0.782)
Random Forest	0.477	0.847 (0.756–0.908)	0.404 (0.276–0.547)	0.778 (0.725–0.829)	0.689 (0.606–0.762)
Gradient Boosting	0.512	0.812 (0.716–0.881)	0.426 (0.295–0.567)	0.762 (0.705–0.817)	0.674 (0.590–0.748)
SVM (RBF)	0.598	0.788 (0.690–0.862)	0.553 (0.412–0.686)	0.775 (0.710–0.832)	0.705 (0.622–0.776)
LightGBM	0.427	0.812 (0.716–0.881)	0.447 (0.314–0.588)	0.767 (0.709–0.822)	0.682 (0.598–0.755)
XGBoost	0.389	0.882 (0.797–0.935)	0.277 (0.169–0.418)	0.773 (0.728–0.816)	0.667 (0.583–0.741)
STACKING	0.409	0.882 (0.797–0.935)	0.404 (0.276–0.547)	0.798 (0.750–0.843)	0.712 (0.630–0.782)

**Table 4:** The 13 features selected by recursive feature elimination (RFE) on the training set, their types, their univariate association with the FTND score and their variance inflation factors. The number of features was fixed a priori at 13; RFE determined which features were retained (Section 2.6). ρ, Spearman’s rank correlation with the FTND score (all *p* < 0.05); VIF, variance inflation factor within the set of 13 features; both computed in the training set (*n* = 528).

Feature	Type	ρ (FTND)	VIF
Smoking duration (years)	Smoking exposure–raw variable	0.37	3.2
Platelet count (PLT)	Raw CBC parameter	0.12	41.5
BMI	Feature engineering–derived index	0.09	18.9
Age × leukocytes (Age × WBC)	Feature engineering–interaction term	0.27	130.5
Age × platelets (Age × PLT)	Feature engineering–interaction term	0.27	31.0
Age × neutrophils (Age × NEU)	Feature engineering–interaction term	0.25	63.7
Age × lymphocytes (Age × LYM)	Feature engineering–interaction term	0.26	18.1
Age × basophils (Age × BASO)	Feature engineering–interaction term	0.16	1.2
Age × eosinophils (Age × EOS)	Feature engineering–interaction term	0.16	1.8
Age × mean corpuscular volume (Age × MCV)	Feature engineering–interaction term	0.26	12.6
Age × plateletcrit (Age × PCT)	Feature engineering–interaction term	0.26	15.7
BMI × hemoglobin (BMI × HGB)	Feature engineering–interaction term	0.09	3.4
BMI × platelets (BMI × PLT)	Feature engineering–interaction term	0.14	52.7

**Table 5:** Results of the ablation analysis of the contribution of the feature groups.

Scenario	Leakage-Free 10-Fold CV AUC
Full feature pool (reference, 49 features)	0.652
(−) Interaction terms removed	0.640
(−) Derived indices removed	0.656
(−) All feature engineering removed (smoking duration retained)	0.667
(−) Smoking duration removed	0.601
No feature engineering (demographic + raw CBC)	0.621
Smoking duration only	0.678
Raw CBC only (17 parameters)	0.594

**Table 6:** Pairwise statistical comparison of the cross-validated AUC values (leakage-free 10-fold CV in the training set, *n* = 528; folds, feature selection and model as in Table 5). The AUCs were computed from the pooled out-of-fold predictions and therefore differ slightly from the fold means in Table 5; ΔAUC (first minus second configuration), its bootstrap 95% CI (3000 stratified resamples) and the DeLong test refer to the pooled AUCs. CI, confidence interval.

Comparison	Pooled Out-of-Fold AUC	ΔAUC	Bootstrap 95% CI (ΔAUC)	DeLong *p*
Full pool (49) vs. smoking duration	0.6537 vs. 0.6743	−0.021	−0.052 to +0.012	0.22
Full pool (49) vs. raw CBC (17)	0.6537 vs. 0.5911	+0.063	+0.010 to +0.113	0.016
Smoking duration vs. raw CBC (17)	0.6743 vs. 0.5911	+0.083	+0.018 to +0.145	0.010

**Table 7:** Sensitivity of the leakage-free 10-fold cross-validated AUC to the FTND cut-off (training set, *n* = 528; for each cut-off, the 80:20 split and the folds were stratified by the corresponding outcome; feature selection and logistic regression as in Table 5). Values are mean ± SD of the ten fold-wise AUCs; the ≥6 row reproduces Table 5. SD, standard deviation.

FTND Cut-Off	Class Balance (High/Low)	Full Pool (49) CV-AUC	Smoking Duration CV-AUC	Raw CBC CV-AUC
≥5	393/135 (74%)	0.732 ± 0.059	0.726 ± 0.071	0.614 ± 0.037
≥6 (primary)	338/190 (64%)	0.652 ± 0.090	0.678 ± 0.066	0.594 ± 0.052
≥7	255/273 (48%)	0.638 ± 0.084	0.653 ± 0.080	0.571 ± 0.042

**Table 8:** SHAP global feature importance ranking (mean |SHAP| value; logistic regression model, training set, *n* = 528).

Feature	Mean |SHAP|
Age × leukocytes (Age × WBC)	0.742
Smoking duration (years)	0.686
Age × neutrophils (Age × NEU)	0.584
Age × platelets (Age × PLT)	0.451
BMI × platelets (BMI × PLT)	0.419
BMI	0.242
Age × lymphocytes (Age × LYM)	0.147
Platelet count (PLT)	0.112
Age × plateletcrit (Age × PCT)	0.091
Age × mean corpuscular volume (Age × MCV)	0.067
Age × basophils (Age × BASO)	0.052
Age × eosinophils (Age × EOS)	0.038
BMI × hemoglobin (BMI × HGB)	0.022

**Table 9:** Spearman rank correlation of the variables with the Fagerström dependence score. Benjamini–Hochberg FDR correction applied. n.s., not significant; * *p* < 0.05; ** *p* < 0.01; *** *p* < 0.001. Outcome-overlapping variable: the number of cigarettes smoked per day is one of the six FTND items (0–3 of the 10 points). Packs per day was therefore excluded from the predictive feature pool, and its correlation with the FTND score is expected by construction; it is shown for reference only and does not constitute independent evidence.

Variable	Spearman ρ	*p* (FDR)	Significance
Smoking duration (years)	+0.353	<0.001	***
Age	+0.247	<0.001	***
Leukocytes (WBC)	+0.184	<0.001	***
Lymphocytes (LYM)	+0.172	<0.001	***
Neutrophils (NEU)	+0.141	0.001	**
Red cell distribution width (RDW)	+0.137	0.002	**
Platelets (PLT)	+0.134	0.002	**
Eosinophils (EOS)	+0.132	0.002	**
Plateletcrit (PCT)	+0.124	0.004	**
Body mass index (BMI)	+0.112	0.010	**
Monocytes (MONO)	+0.103	0.017	*
Basophils (BASO)	+0.089	0.041	*
Mean corpuscular hemoglobin concentration (MCHC)	+0.049	0.308	n.s.
Weight	+0.046	0.334	n.s.
Mean corpuscular hemoglobin (MCH)	+0.036	0.441	n.s.
SII	+0.032	0.483	n.s.
Hemoglobin (HGB)	+0.027	0.547	n.s.
Mean corpuscular volume (MCV)	+0.012	0.820	n.s.
Hematocrit (HCT)	+0.010	0.827	n.s.
Erythrocytes (RBC)	+0.001	0.979	n.s.
NLR	−0.036	0.441	n.s.
MLR	−0.040	0.415	n.s.
PLR	−0.062	0.175	n.s.
Mean platelet volume (MPV)	−0.081	0.064	n.s.
Platelet distribution width (PDW)	−0.088	0.041	*
Height	−0.103	0.017	*
Outcome-overlapping variable (reference only)			
Packs per day (Packs/day)	+0.551	<0.001	***

## Data Availability

The de-identified dataset analyzed during the current study is available from the corresponding author, Yavuz Korkmaz, upon reasonable request and with the permission of the Scientific Research Ethics Committee for Health Sciences of Malatya Turgut Özal University. The data are not publicly available because they were derived from hospital medical records under institutional data-protection restrictions. Owing to ethical restrictions associated with the clinical dataset, the code used for data preprocessing, feature engineering, feature selection, model training, evaluation and interpretability analyses is not publicly available; it can be obtained from the corresponding author, Yavuz Korkmaz, upon reasonable request.

## Supplementary Materials

The following supporting information can be downloaded at: https://www.mdpi.com/article/10.3390/diagnostics16203425/s1, Figure S1: Calibration of the seven models on the hold-out test set; Figure S2: Feature importance values of the Random Forest model; Figure S3: LIME local explanation of the patient with the highest predicted probability of High dependence (*p* = 0.875); Figure S4: LIME local explanation of the patient with the lowest predicted probability of High dependence (*p* = 0.208).

## Abbreviations

The following abbreviations are used in this manuscript:

AUC	Area under the curve
BASO	Basophils
BMI	Body mass index
CBC	Complete blood count
CI	Confidence interval
CV	Cross-validation
EEG	Electroencephalography
EOS	Eosinophils
FDR	False discovery rate
FTND	Fagerström Test for Nicotine Dependence
FTQ	Fagerström Tolerance Questionnaire
HCT	Hematocrit
HGB	Hemoglobin
LIME	Local Interpretable Model-agnostic Explanations
LMR	Lymphocyte-to-monocyte ratio
LYM	Lymphocytes
MAE	Mean absolute error
MCH	Mean corpuscular hemoglobin
MCHC	Mean corpuscular hemoglobin concentration
MCV	Mean corpuscular volume
MLR	Monocyte-to-lymphocyte ratio
MONO	Monocytes
MPV	Mean platelet volume
MRI	Magnetic resonance imaging
NEU	Neutrophils
NLR	Neutrophil-to-lymphocyte ratio
PCT	Plateletcrit
PDW	Platelet distribution width
PLR	Platelet-to-lymphocyte ratio
PLT	Platelets
RBC	Erythrocytes
RBF	Radial basis function
RDW	Red cell distribution width
RFE	Recursive feature elimination
RMSE	Root mean squared error
ROC	Receiver operating characteristic
SD	Standard deviation
SHAP	SHapley Additive exPlanations
SII	Systemic immune-inflammation index
SVM	Support vector machine
VIF	Variance inflation factor
