# Supplementary material for: Modeling Factors Associated with Smoking Dependence Severity Using Binary Classification-Based Machine Learning: Prediction with Hematological and Demographic Patient Data
Source: Diagnostics (Basel). 2026 Oct 21;16(20):3425. doi: 10.3390/diagnostics16203425 (PMC13702976; doi:10.3390/diagnostics16203425)
Supplement: Supplementary file 1 [file diagnostics-16-03425-s001.zip › diagnostics-4581355-supplementary.pdf]

Supplementary Materials

Modeling Factors Associated with Smoking Dependence Severity Using Binary Classification-Based Machine Learning: Prediction with Hematological and Demographic Patient Data

Yavuz Korkmaz, İbrahim Miraç Eligüzel and Adem Taha Özdemir

Diagnostics, manuscript ID diagnostics-4581355

Contents

Figure S1. Calibration of the seven models on the hold-out test set.

Figure S2. Feature importance values of the Random Forest model.

Figure S3. LIME local explanation of the patient with the highest predicted probability of High dependence (P = 0.875).

Figure S4. LIME local explanation of the patient with the lowest predicted probability of High dependence (P = 0.208).

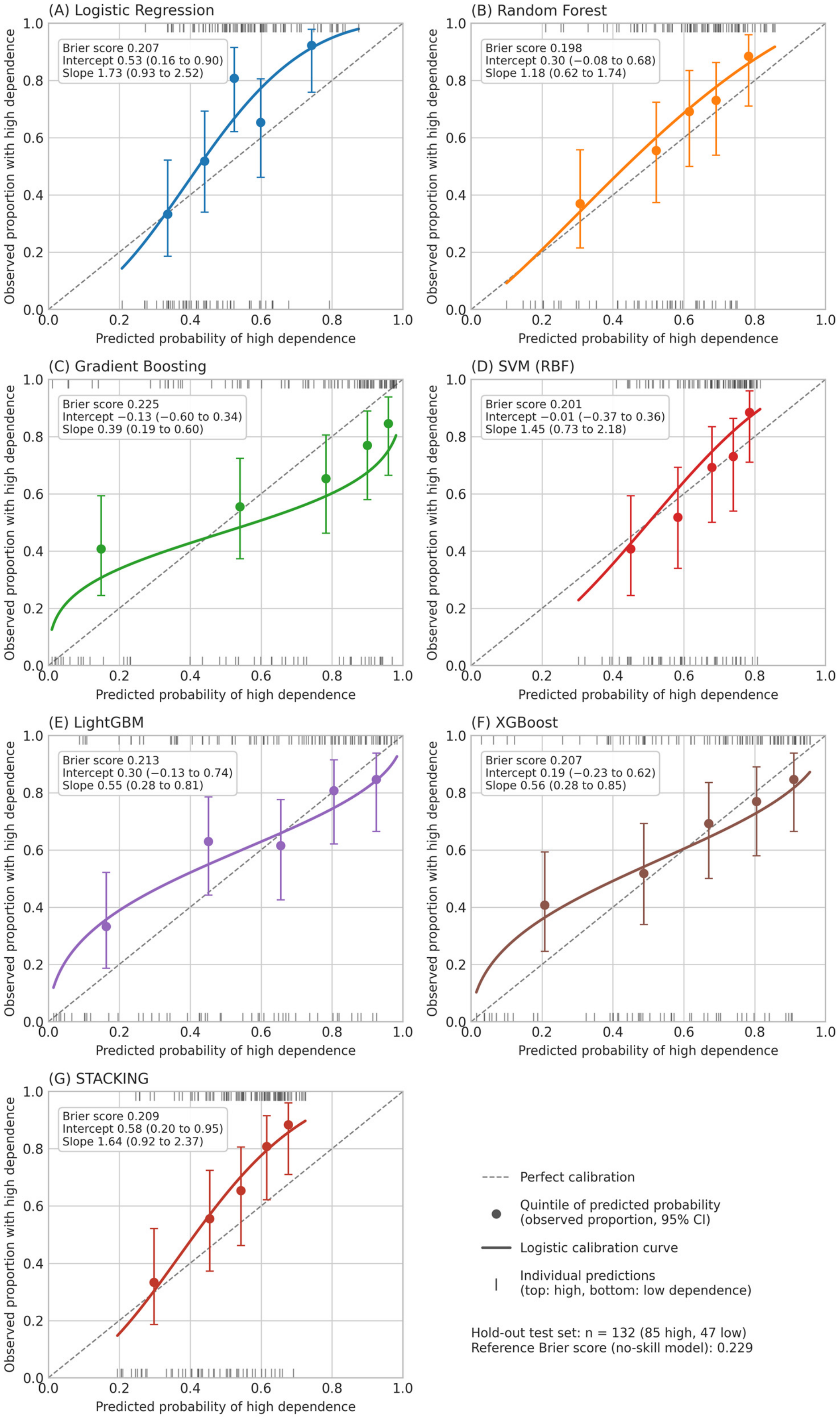

Figure S1. Calibration of the seven models on the hold-out test set (n = 132; 85 High, 47 Low). Points: observed proportion of high dependence (FTND ≥ 6) in each quintile of predicted probability (26–27 patients per quintile), with Wilson 95% confidence intervals, plotted against the mean predicted probability; solid line: logistic calibration curve; dashed line: perfect calibration; tick marks: individual predicted probabilities (top: high dependence; bottom: low dependence). Each panel gives the Brier score and the calibration intercept and slope with their 95% confidence intervals; the Brier score of a non-informative reference model is 0.229.

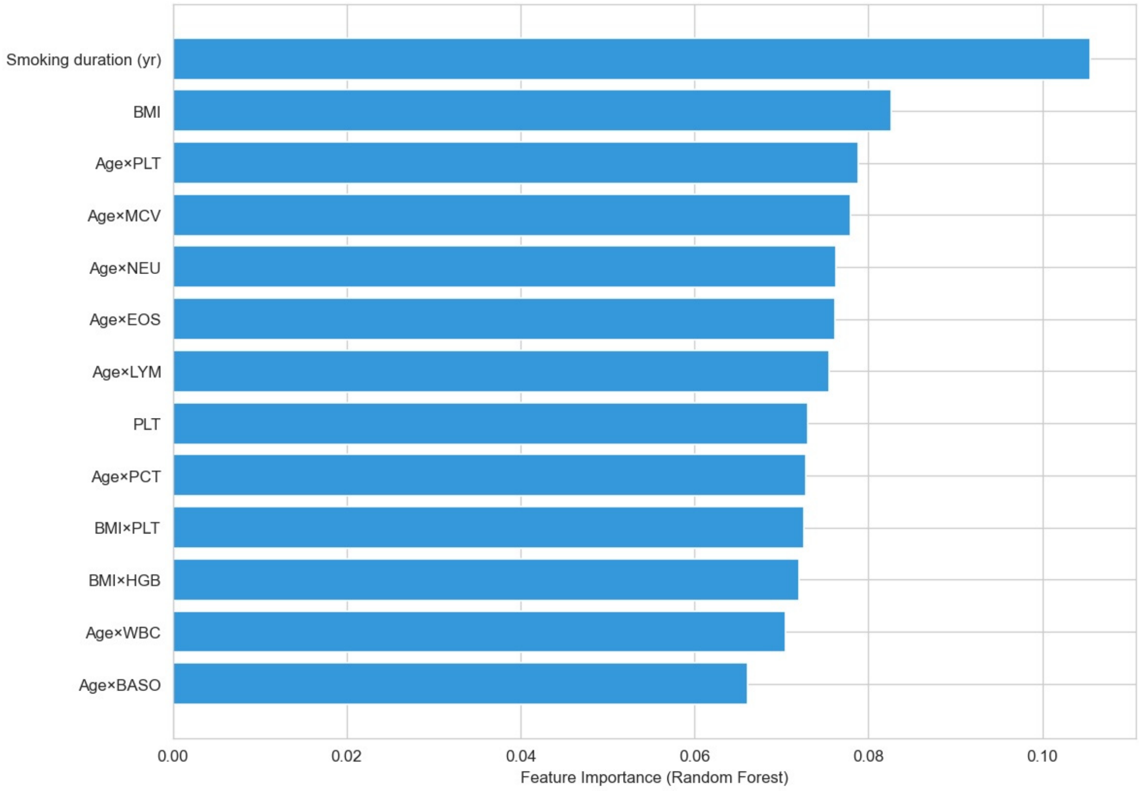

Figure S2. Feature importance values of the Random Forest model (impurity-based importance; training set, n = 528).

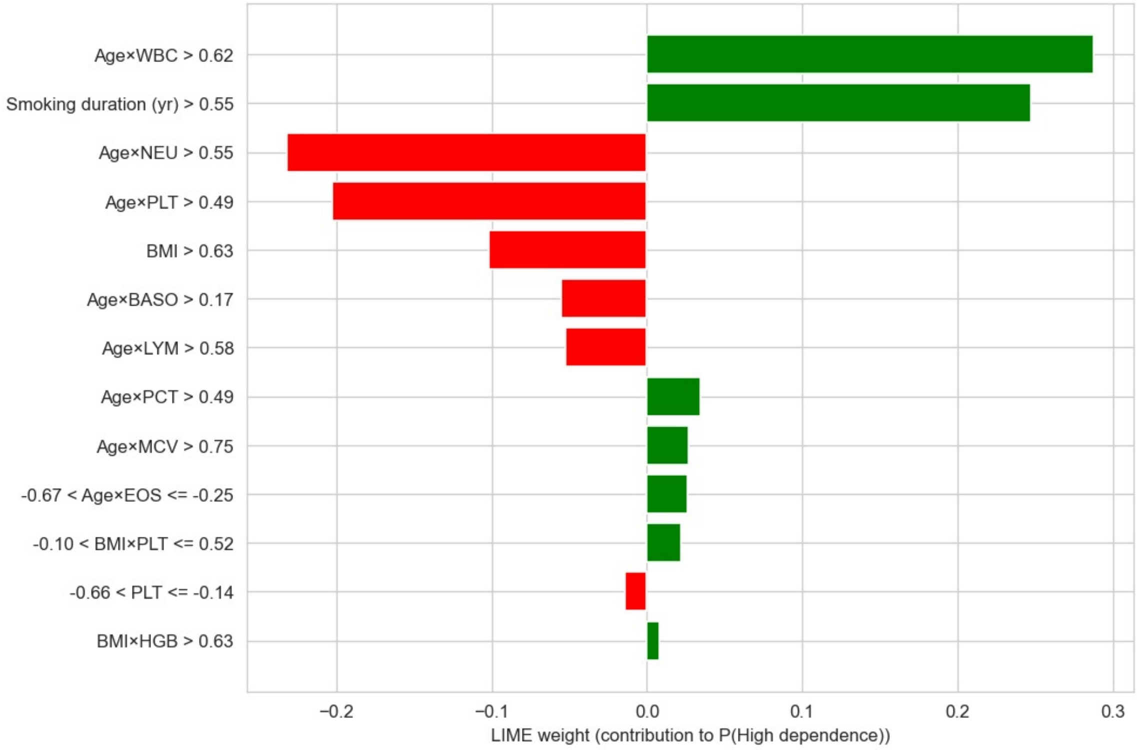

Figure S3. LIME local explanation — patient for whom the probability of High dependence was predicted as P = 0.875 (true class: High) (logistic regression model; training set, n = 528).

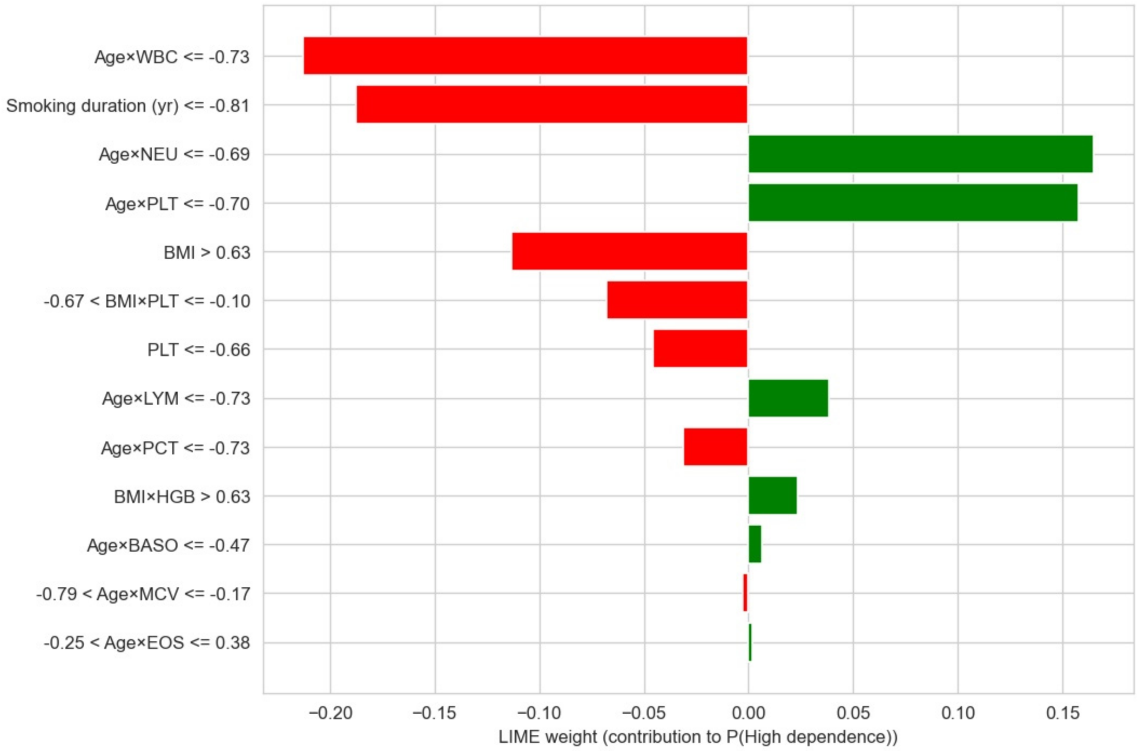

Figure S4. LIME local explanation — patient for whom the probability of High dependence was predicted as P = 0.208 (true class: Low) (logistic regression model; training set, n = 528).
